# First time exploration and characterization of key-intermediates in palladium-catalysed coupling reactions

**DOI:** 10.1038/s41598-026-43634-1

**Published:** 2026-03-18

**Authors:** Péter Szuroczki, Attila Bényei, Rafael T. Aroso, Mariette M. Pereira, László Kollár

**Affiliations:** 1 Department of Inorganic Chemistry, University of Pécs, Ifjúság Útja 6., P.O.Box 266, 7624 Pécs, Hungary; 2 János Szentágothai Research Centre, University of Pécs, Ifjúság Útja 20., 7624 Pécs, Hungary; 3 HUN-REN-PTE Selective Chemical Synthesis Research Group, Ifjúság Útja 6., 7624 Pécs, Hungary; 4 Department of Physical Chemistry, University of Debrecen, Egyetem Tér 1., Debrecen, 4032 Hungary; 5 Coimbra Chemistry Centre, Department of Chemistry, University of Coimbra, Rua Larga, 3004-535 Coimbra, Portugal

**Keywords:** Palladium, Porphyrin, Phosphine, Oxidative addition, X-ray crystallography, Biochemistry, Chemistry

## Abstract

The oxidative addition of a bromoporphyrin onto in situ formed highly active Pd(0) complex allowed us to characterize the Pd(II) intermediate by X-ray crystallography. In addition, after the heterobidentate coordination of Xantphos hemioxide (*Xantphos:* 4,5-bis(diphenylphosphino)-9,9-dimethyl-xanthene), the oxidized product responsible for Pd(II)-Pd(0) reduction, was also proved. The species isolated by us is the product of oxidative addition, the first step of the suggested catalytic cycle. Earlier only the structure of the dichloro complex of palladium with (P-P(O)) ligand was known. The isolated Pd(P-P(O))X(porphyrinyl) complex can be considered as a new example of catalytic intermediates of important cross-coupling and carbonylation reactions giving further strong proof for their mechanism.

**Supplementary Information:**

The online version contains supplementary material available at 10.1038/s41598-026-43634-1.

## Introduction

The role of oxidative addition in transition metal chemistry cannot be overstated, given its central relevance in catalytic processes.^1–3^ The most important features include the increase in coordination number and oxidation state of the metal center, as well as a two-electron gain, which are thoroughly discussed in reviews and textbooks.^4–9^ As the first step in many catalytic cycles, oxidative addition has received particular attention in the 1970s and extensive mechanistic information had already been gathered on the role of low-valent transition metals (Ni(0), Pd(0)) with substrates such as aryl and benzyl halides.^10,11^

In the context of the Heck–Mizoroki^12,13^ and related cross-coupling reactions, Knowles and Whiting provided a detailed analysis of oxidative addition to Pd(0).^14^ It´s also well established that substrate activation in homogeneous, transition metal-catalyzed reactions proceeds through diverse pathways, depending on the electronic structures of both the metal center and the substrate. Generally, nonpolar substrates undergo oxidative addition via concerted mechanisms, yielding complexes where the new ligands occupy *cis* positions, typical of homogeneous hydrogenation and hydroformylation.^15–18^ In contrast, polar or electrophilic substrates react stepwise, forming *trans* intermediates in successive elementary steps. Cross-coupling reactions (Heck, Suzuki, Negishi, Kumada, Stille, Sonogashira) and their carbonylative variants, which revolutionized modern synthesis, follow this later stepwise pathway.^19–23^ In Pd-catalyzed cross-coupling, oxidative addition involves insertion of an aryl or alkenyl halide (or a sulfonate surrogate) into an in situ generated Pd(0)-phosphine species.^24,25^ It is worth noting that Ni(0) and Pt(0) precurzors of lower catalytic importance regarding the above reactions, have also been reacted with similar bromaromatics in oxidative addition.^26–30^ In general the Pt(II) complexes formed are kinetically less labile than the corresponding Pd-complexes.

**Fig. 1:**
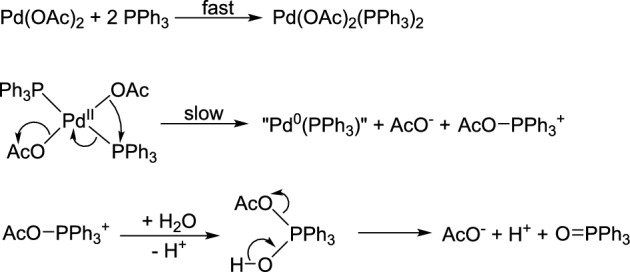
Formation of Pd(0) complexes from Pd(OAc)_2_ in the presence of PPh_3_ as reducing agent^31,32^.

As for the highly active palladium systems, both preformed Pd(0) precursors (Pd(PPh₃)₄), Pd_2_(dba)_3_) and Pd(II) sources (Pd(OAc)₂), PdCl_2_) can be used. It has also been shown that Pd(II) is reduced to Pd(0) by tertiary phosphines. Cyclic voltammetry studies revealed that PPh₃ is oxidized to triphenylphosphine oxide while the highly reactive, coordinatively unsaturated 14-electron Pd(0)-monophosphine species (indicated as ‘Pd(PPh_3_)(L)’, or in the presence of good donor solvents, ‘Pd(PPh_3_)(solvent)’) is formed (Fig. 1).^31,32^

Although monodentate phosphines dominate most cross-coupling protocols, bidentate ligands exhibit significant advantages.^33,34^ The key difference lies in geometry: with monodentate ligands, the oxidative addition product is usually *trans*, whereas bidentate chelates impose a *cis* configuration which is essential for efficient reductive elimination. However, the oxidation of bidentate phosphines during Pd(II) reduction generates “monodentate” phosphine–phosphine oxide ligands containing both P(III) and P(V) centers. NMR studies have supported the in situ formation of diphosphine hemi-oxides in Pd(II)-acetate/diphosphine systems.^35^

These phosphine–phosphine oxide ligands, although potentially capable of P,O-hemilable bidentate coordination, are generally regarded as monodentate. Consequently, statements such as “*it is necessary to use at least two equivalents of a bidentate phosphine*” are frequently encountered in the literature.^14^ This assumption, however, introduce both mechanistic and synthetic complications arising from the coexistence of mono- and dinuclear palladium species with mixed mono- and bidentate coordination modes.^19–23,36^ It is worth noting that the (partial) oxidation of diphosphines can be avoided by employing Pd(0) precursors such as tris(dibenzylideneacetone)dipalladium(0), (Pd₂(dba)₃) or Pd(dba)₂. However, in these systems, the coordinating dba ligand may inhibit the reaction by hindering oxidative addition.^37^

Regarding ligand selection, the seminal studies by van Leeuwen et al*.* on large bite-angle diphosphines are particularly noteworthy.^38–41^ The discovery of the *Xantphos* ligand family, which exhibit bite angles of 100–120°, had a profound impact on numerous transition metal-catalyzed reactions.^42^ As for carbonylation reactions, the work of Buchwald et al.on aryl bromide carbonylation with palladium-Xantphos in situ system has to be mentioned.^43^ Recently, highly active and selective Pd–Xantphos catalysts have been applied to aminocarbonylation (Heck–carbonylation) of haloaromatic substrates in our laboratory.^44,45^ Despite being repeatedly proposed as crucial intermediates in cross-coupling and carbonylation catalysis, Pd(diphosphine hemioxide)(halide)(aryl) complexes have never been experimentally observed or isolated**.** It is crucial, that herein, we report the first isolation and X-ray characterization of an in situ formed Pd(II)–Xantphos hemioxide complex containing a halide and a substrate (porphyrinyl) as ligands. This finding provides direct evidence for the heterobidentate coordination of the hemilabile P–P(O) ligand to a Pd(0) species (generated from a Pd(II) precursor) and for the oxidative addition of a bromo-porphyrin substrate to the Pd(0) center, thus revealing, for the first time, the structure of a long-hypothesized catalytic intermediate.

## Results and discussion

**Fig. 2:**
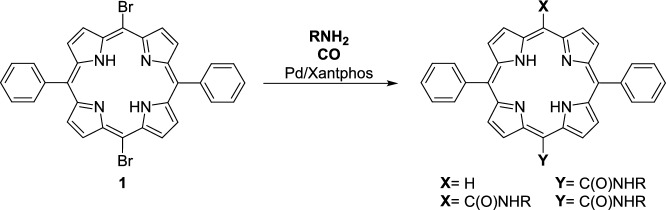
A general scheme for the aminocarbonylation of **DPP-Br**_**2**_** (1)**^49^.

**Fig. 3:**
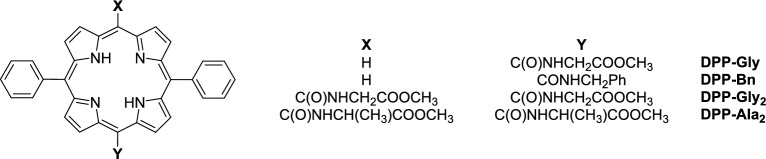
Porphyrin monocarboxamides (**DPP-Bn** and **DPP-Gly**) and dicarboxamides (**DPP-Gly**_**2**_ and **DPP-Ala**_**2**_) characterized by X-ray crystallography (for detailed information See SI Figures S1-4).

As recently reported by us, a series of carboxamide-functionalised diphenylporphyrins (DPPs) with unprecedented structural features have been synthesized in good yields via palladium-catalysed carbonylation^49^ (*Heck–carbonylation*^46–48^). Under standard aminocarbonylation conditions (1 bar CO, 70 °C, Pd(OAc)₂ as catalyst precursor and 1 equivalent of Xantphos as ligand), several DPP-based mono- and dicarboxamides were obtained from **DPP–Br₂ (1)** as the substrate (Fig. 2).^49^ In addition, we have also been able to get a single-crystal of four of the porphyrin-based carboxamides, **DPP-Bn**, **DPP-Gly**, **DPP-Gly**_**2**_ and **DPP-Ala**_**2**_ (Fig. 3) and determine their structure by X-ray crystallography (See SI Fig. S1-4).

It is important to note that the Pd/Xantphos ratio of 1/1 was used throughout the catalytic investigations because of the following reasons: i) A perfectly homogeneous catalytic mixture was obtained even at the above low ratio of 1/1. ii) A highly active catalytic system was obtained and the addition of a second equivalent of Xantphos did not improve catalytic performance. In turn, previous investigations have shown that the use of Xantphos in twofold (or higher) excess has a deteriorative effect on activity.^50^ iii) The presence of diphosphine, as well as its dioxide and hemioxide in the final reaction mixture makes the work-up procedure, *i.e*., the isolation of the target product, more difficult.

From the catalytic reaction mixture of palladium catalysed carbonylation, using L-alanine methyl ester hydrocloride as nucleophile and DPP–Br₂ **(1)** as substrate, a crystalline chloro-porphyrinyl-palladium(II) complex (**3**) was formed and isolated.

**Fig. 4:**
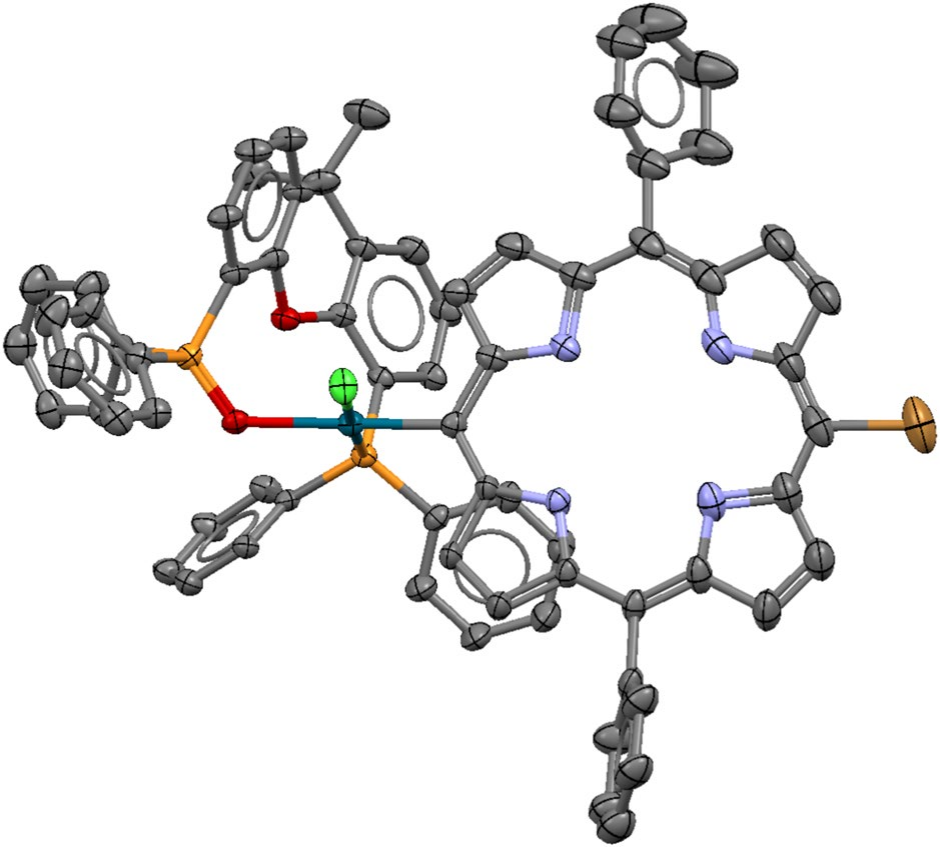
X-ray crystal structure of **3.** ORTEP view at 40% probability level, hydrogen atoms are omitted for clarity.

The compound has been fully characterised including single-crystal X-ray crystallography (Fig. 4 and SI).

The structure of P-P(O) palladium complex **3** is remarkable and unprecedented. A search of the Cambridge Structural Database (Version 6.0, Update April 2025)^51^ revealed only one structurally related palladium complex (PdCl_2_(P-P(O)), ref. code WIGDOJ)^52^ and one zinc complex **(**ZnCl_2_(P-P(O)), ref. code VELPUB),^53^ both being dichloro complexes of the Xantphos hemioxide (P-P(O)) ligand**.** In our case, however, the chlorine atom *trans* to the coordinated oxygen is replaced by the porphyrin *meso* carbon atom (C15)**,** allowing, for the first time, the structural characterization of a key intermediate in the catalytic cycle**.** Aside from this unique feature, the palladium centre adopts a typical square-planar coordination geometry, as evidenced by the bond distance and angle data (Table S3).

**Fig. 5:**
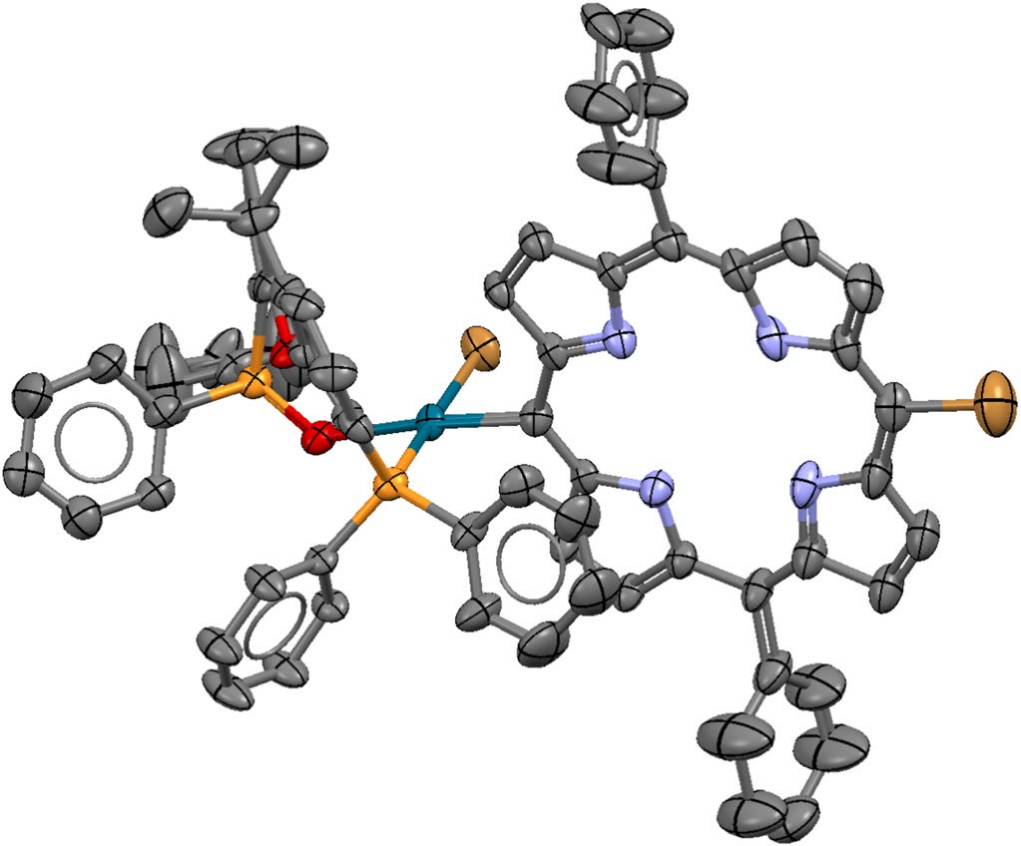
X-ray crystal structure of **2**, ORTEP view at 40% probability level, solvent chloroform as well as water molecule within the porphyrin ring and hydrogen atoms are omitted for clarity (only one enantiomer is shown).

The following further features need to be emphasized. Although Pd(OAc)_2_/Xantphos in situ catalyst was used, the complex contains the hemioxide of Xantphos (abbreviated as P-P(O) in further discussion) coordinated as a hemilabile heterobidentate ligand. Its formation can be explained by the oxidation of the Xantphos diphosphine during the Pd(II)-Pd(0) reduction. While Pd(OAc)_2_ is reduced to a coordinatively highly unsaturated Pd(0) complex, one of the two phosphorus of the bidentate ligand is oxidised to phosphine oxide. Although the bidentate coordination of diphosphine hemioxide to Pd(II) has already been described,^54,55^ to the best of our knowledge, it is the first time to prove its presence in a catalytically active species (Fig. 5).

**Fig. 6:**
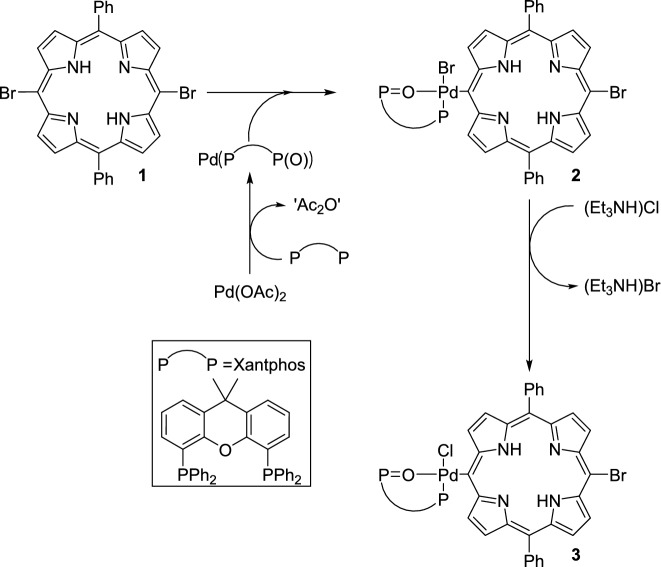
Oxidative addition of **1** onto palladium(0) species formed in situ from Pd(OAc)_2_ and Xantphos.

According to a generally accepted mechanism of aminocarbonylation,^56–67^ the oxidative addition of **1** onto in situ formed Pd(0) complex should have resulted in the formation of a bromo-porphyrinyl-palladium(II) complex (**2**). However, the triethylammonium chloride salt, formed in the dehydrochlorination of methyl alaninate hydrochloride, acts as chloride source in bromo-chloro ligand exchange yielding **3** under catalytic conditions (Fig. 6).

The above reaction sequence was proved by carrying out an independent experiment aiming at the direct synthesis of the bromo complex (**2**). In fact, reacting **1** with the in situ generated Pd(0) complex in the absence of a primary amine nucleophile, complex **2** was obtained. The efforts on crystallization led to good-quality needle-like crystals suitable for single-crystal X-ray crystallography which allowed us to unequivocally establish the molecular and crystal structure of the bromo-complex **2** (Fig. 5).

**Fig. 7:**
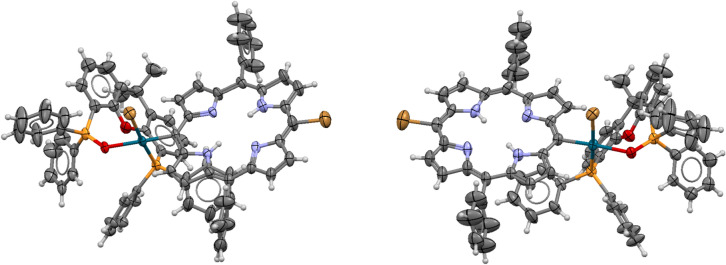
The two PdBr(Porph)(P-P(O)) enantiomers found in the asymmetric unit of structure **2.** Solvent chloroform and water molecules within the porphyrine moieties are omitted for clarity.

The structure of **2** is even more peculiar than that of **3**. In this case the compound crystallized in a space group allowed for enantiomer pure compounds (monoclinic P2_1_, Z = 4). We have a racemate, and the two enantiomers are in the asymmetric unit (Z’ = 2, Fig. 7) but at the same time the crystal is a racemic twin and that is indicated by the Flack parameter which is 0.5 within experimental error (F = 0.43). Compound **3** is also chiral and racemate, but in that case the space group is centrosymmetric (triclinic, P-1, Z = 2) with one enantiomer in the asymmetric unit (Z’ = 1) and the other enantiomer is generated by the lattice symmetry. The bond distance data for **2** and **3** (*Table S3*) are also reasonable for the P(O)-P ligand coordination.

**Fig. 8:**
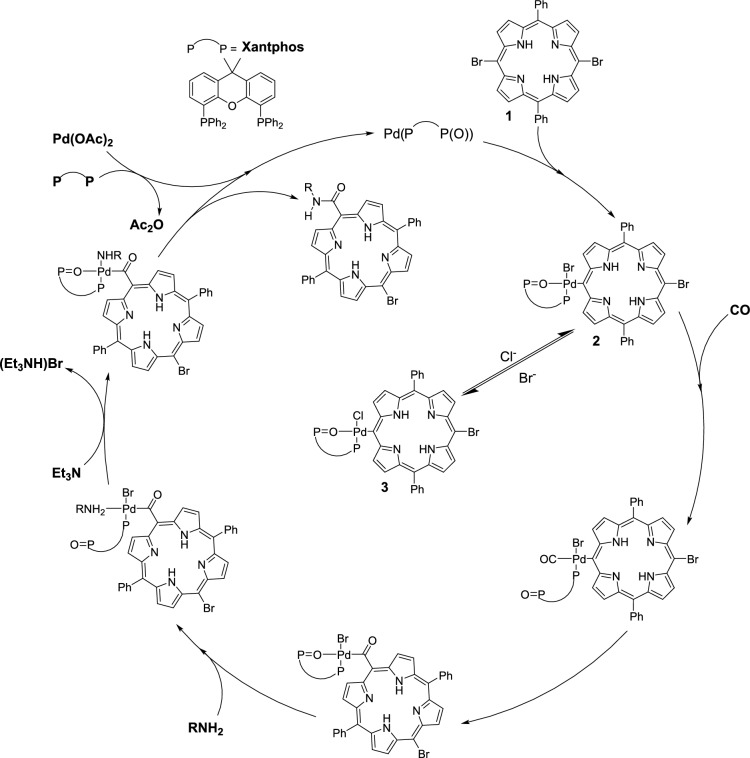
A proposed catalytic cycle of palladium-catalysed aminocarbonylation using bromoporphyrin as substrate and Xantphos as ligand.

Although only the oxidative addition (the ‘starting step’ of aminocarbonylation) was discussed above, it can be embedded in a widely accepted catalytic cycle (Fig. 8). The formation of carboxamides can be rationalised based on generally accepted sequence of steps such as oxidative addition of the substrate on Pd(P-P(O)), carbon monoxide activation as a terminal carbonyl ligand, its insertion into the palladium-carbon bond forming an acyl complex, coordination of the amin, the loss of hydrogen halide resulting in an amide-acyl complex, and reductive elimination in the closing (‘product-forming’) step.

## Methods

### General procedures

The Pd(OAc)_2_, Xantphos (4,5-bis(diphenylphosphino)-9,9-dimethylxanthene)), Et_3_N, all solvents, amines were purchased from Sigma-Aldrich (St. Louis, MO, USA) and were used without any further purification. The starting material (**1**) was prepared according to the reported protocol.^68,69^ All reactions were carried out under Ar using standard Schlenk techniques.

Precoated silica gel 60F_254_ plates were used for thin layer chromatography (TLC) and were also purchased from Sigma-Aldrich. Column chromatography was performed with 0.063–0.200 mm mesh silica gels. ^1^H, ^13^C and ^31^P NMR spectra were recorded in CDCl_3_ or DMSO-d_6_ on a Bruker Avance III 500 spectrometer (Bruker BioSpin Corp., Karlsruhe, Germany) at 500, 126 and 200 MHz, respectively. Chemical shifts δ are reported in ppm relative to CDCl_3_ (7.26 and 77.00 ppm for ^1^H and ^13^C, respectively), DMSO-d_6_ (2.50 and 39.50 ppm for ^1^H and ^13^C, respectively) or H_3_PO_4_ (85%) (0.00 ppm for ^31^P). The FT-IR spectra were taken in KBr pellets using a Nicolet IMPACT 400 spectrometer (Thermo Fisher Scientific, Waltham, MA, USA) applying a DTGS detector in the region of 400–4000 cm^-1^, the resolution was 4 cm^-1^.

High-resolution mass spectra were acquired on a 6530 Accurate-Mass Quadrupole Time-of-Flight (Q-TOF) LC/MS system (Agilent Technologies, Singapore) equipped with an Agilent Jet Stream electrospray ionization (ESI) source.

### X-ray crystallographic study

The unambiguous molecular structures of **2** and **3** have been established by X-ray diffraction analysis. X-ray quality crystals of both compounds were grown from the slow evaporation of concentrated solutions of *n*-hexane/chloroform standing at ambient temperature. A properly chosen suitable crystal was then fixed under a microscope onto a Mitegen loop using high-density oil. Diffraction Intensity data was collected at ambient temperature (294 K) on a Bruker-D8 Venture diffractometer (Bruker AXS GmbH, Karlsruhe, Germany) equipped with INCOATEC IµS 3.0 (Incoatec GmbH, Geesthacht, Germany) dual (Cu and Mo) sealed tube micro sources and a Photon II Charge-Integrating Pixel Array detector (Bruker AXS GmbH, Karlsruhe, Germany) using Mo Kα (λ = 0.71073 Å) radiation.

High-multiplicity data collection and integration were performed using APEX3 (version 2017.3–0, Bruker AXS Inc., 2017, Madison, WI, USA) software. Data reduction and multiscan absorption correction were performed using SAINT (version 8.38A, Bruker AXS Inc., 2017, Madison, WI, USA). The structure was solved using direct methods and refined on F^2^ using the SHELXL program^70^ incorporated into the APEX3 suite. Refinement was performed anisotropically for all non-hydrogen atoms. Hydrogen atoms were placed in idealized positions on parent atoms in the final refinement including adjacent hydrogen atoms within the porphyrin ring.

The CIF file was manually merged using publCIF software,^71^ while graphics were designed using the Mercury program.^72^ The results for the X-ray diffraction structure determinations followed the Checkcif functionality of PLATON software (Utrecht University, Utrecht, the Netherlands),^73^ and structural parameters, such as bond length and angle data, are in the expected range. The DPP compounds had high tendency to form twin crystals and in some cases the size of the crystals were very small, thin plate or needle. These features resulted in A and B level errors concerning the R_int_ and final R error data. However, all of the structures are considered to be correct and high importance in the field. Further details of the crystal parameters, data collection, and structure refinement are given in Table S1 and Table S2. The geometric parameters for all structures are given in Tables S3-S9.

The datasets generated and/or analysed during the current study are available in the Crystallographic Data Centre repository, http://www.ccdc.cam.ac.uk/data_request/cif using reference deposition numbers: 2512487 for **2** and 2512488 for **3** (Pd-complexes), as well as 2512489 for **DPP-Bn,** 2512490 for **DPP-Gly,** 2385880 for **DPP-Gly2** and 2512491 for **DPP-Ala2** (porphyrin carboxamides).

## Conclusion

Most of the palladium-catalysed carbonylation and coupling reactions proceed via palladium(0) species enabling the activation of the substrate by its oxidative addition. In this work, the use of a bromoporphyrin substrate allowed us to characterize the porphyrin-Pd(II) intermediate by X-ray crystallography. In addition, the formation and heterobidentate coordination of Xantphos hemioxide, the oxidized product responsible for Pd(II)-Pd(0) reduction, was also proved. The isolated Pd(P-P(O))X(porphyrinyl) complexes can be considered as fully characterised new examples of catalytic intermediates and allow us to understand the nature of active catalysts.

## Supplementary Information

Supplementary Information.

## Data Availability

The datasets generated and/or analysed during the current study are available in the Crystallographic Data Centre repository, http://www.ccdc.cam.ac.uk/data_request/cif using reference deposition numbers: 2512487 for 2 and 2,512,488 for 3, as well as 2,512,489 for DPP-Bn, 2,512,490 for DPP-Gly, 2,385,880 for DPP-Gly2 and 2,512,491 for DPP-Ala2. In addition to analytical data (1H and 13C NMR, MS) discussed in Supplementary Information, further details are available from the authors upon request.
