# Supplementary material for: First time exploration and characterization of key-intermediates in palladium-catalysed coupling reactions
Source: Sci Rep. 2026 Mar 18;16:14059. doi: 10.1038/s41598-026-43634-1 (PMC13136337; doi:10.1038/s41598-026-43634-1)
Supplement: Supplementary file 1 — Supplementary Information. [file 41598_2026_43634_MOESM1_ESM.docx]

**Supplementary Information**

to the manuscript

**First Time Exploration and Characterization of Key-intermediates in Palladium-catalysed Coupling Reactions.**

Péter Szuroczki a,b), Attila Bényei c), Rafael T. Aroso d), Mariette M. Pereira *d) and László Kollár *a,b)

1. *Department of Inorganic Chemistry, University of Pécs, Ifjúság útja 6., H-7624 Pécs, Hungary and János Szentágothai Research Centre, University of Pécs, Ifjúság útja 20., H-7624 Pécs, Hungary*
2. *HUN-REN-PTE Selective Chemical Synthesis Research Group, Ifjúság útja 6., H-7624 Pécs, Hungary*
3. *Department of Physical Chemistry, University of Debrecen, Egyetem tér 1., Debrecen, H-4032, Hungary*
4. *Coimbra Chemistry Centre, Department of Chemistry, University of Coimbra, Rua Larga, 3004-535, Coimbra, Portugal*

*Content*

Table S1. Crystal parameters, data collection, and structure refinement details, DPP compounds page 2

Table S2. Crystal parameters, data collection, and structure refinement details, Palladium complexes **2** and **3**  page 3

Table S3 Selected distance (Å), angle data for PdX(P-P(O)) moieties page 4

Table S4. Geometric parameters for **DPP-Bn** page 4

Table S5. Geometric parameters for **DPP-Gly** page 8

Table S6. Geometric parameters for **DPP-Gly2** page 12

Table S7. Geometric parameters for **DPP-Ala2** page 15

Table S8. Geometric parameters for **2** page 20

Table S9. Geometric parameters for **3** page 31

Figure S1. ORTEP view of **DPP-Bn** page 37

Figure S2. ORTEP view of **DPP-Gly** page 38

Figure S3. ORTEP view of **DPP-Gly2** page 38

Figure S4. ORTEP view of **DPP-Ala2** page 39

Palladium-catalysed aminocarbonylation of **DPPBr2** (**1**) page 39

Synthesis of **2** page 40

Characterization of **2** (1H, 13C and 31P NMR, MS, IR) page 40

Characterization of **3** page 47

Table S1. Crystal parameters, data collection, and structure refinement details, **DPP** compounds

| **Compound** | **DPP-Bn** | **DPP-Gly** | **DPP-Ala2** | **DPP-Gly2** |
| --- | --- | --- | --- | --- |
| Crystal data | | | | |
| Chemical formula | C40H29N5O | C36H27N5O3·CHCl3 | C42H36N6O6 | C40H32N6O6·2(CHCl3) |
| *M*r | 595.68 | 696.99 | 720.77 | 931.45 |
| Crystal system, space group | Triclinic, *P*¯1 | Triclinic, *P*¯1 | Orthorhombic, *P*212121 | Monoclinic, *P*21/*c* |
| Temperature (K) | 150 | 293 | 296 | 297 |
| *a*, *b*, *c* (Å) | 9.932 (9), 10.083 (10), 14.647 (14) | 9.775 (7), 10.099 (8), 16.745 (14) | 9.130 (3), 14.595 (5), 27.762 (9) | 7.494 (3), 23.215 (7), 12.347 (4) |
| α, β, γ (°) | 85.12 (4), 83.75 (4), 87.30 (3) | 94.61 (4), 102.00 (4), 90.85 (4) | 90, 90, 90 | 90, 101.494 (11), 90 |
| *V* (Å3) | 1452 (2) | 1611 (2) | 3699 (2) | 2105.0 (11) |
| *Z* | 2 | 2 | 4 | 2 |
| Radiation type | Mo *K*α | | | |
|  (mm-1) | 0.08 | 0.33 | 0.09 | 0.46 |
| Crystal size (mm) | 0.37 × 0.18 × 0.10 | 0.28 × 0.22 × 0.10 | 0.15 × 0.09 × 0.04 | 0.77 × 0.08 × 0.04 |
|  | | | | |
| Data collection | | | | |
| Diffractometer | Bruker D8 VENTURE | | | |
| Absorption correction | Multi-scan Krause, L., Herbst-Irmer, R., Sheldrick, G. M., Stalke, D. (2015). "Comparison of silver and molybdenum microfocus X-ray sources for single-crystal structure determination" J. Appl. Cryst. 48, 3-10. doi:10.1107/S1600576714022985 | | | |
| *T*min, *T*max | 0.43, 0.99 | 0.46, 0.97 | 0.81, 1.00 | 0.52, 0.98 |
| No. of measured, independent and  observed [*I* > 2σ(*I*)] reflections | 9847, 2659, 1589 | 14210, 3436, 1853 | 21480, 2923, 2149 | 62835, 4355, 3254 |
| *R*int | 0.190 | 0.197 | 0.105 | 0.107 |
| max (°) | 20.0 | 21.3 | 18.9 | 26.5 |
| (sin /λ)max (Å-1) | 0.480 | 0.512 | 0.456 | 0.628 |
|  | | | | |
| Refinement | | | | |
| *R*[*F*2 > 2σ(*F*2)], *wR*(*F*2), *S* | 0.103, 0.281, 1.02 | 0.096, 0.263, 1.01 | 0.060, 0.140, 1.02 | 0.078, 0.228, 1.03 |
| No. of reflections | 2659 | 3436 | 2923 | 4355 |
| No. of parameters | 419 | 444 | 498 | 279 |
| No. of restraints | 1 | 3 | 2 | 2 |
| H-atom treatment | H atoms treated by a mixture of independent and constrained refinement | | | |
| max, min (e Å-3) | 0.87, -0.35 | 0.86, -0.31 | 0.14, -0.16 | 1.95, -0.76 |

Table S2. Crystal parameters, data collection, and structure refinement details, palladium complexes **2** and **3**.

| **Compound** | **2** | **3** |
| --- | --- | --- |
| Crystal data | | |
| Chemical formula | C71H54Br2N4O3P2Pd·CHCl3 | C71H52BrClN4O2P2Pd |
| *M*r | 1458.71 | 1276.86 |
| Crystal system, space group | Monoclinic, *P*21 | Triclinic, *P*¯1 |
| Temperature (K) | 289 | 296 |
| *a*, *b*, *c* (Å) | 11.0750 (15), 27.981 (6), 20.767 (3) | 10.977 (3), 15.058 (5), 18.255 (7) |
| α, β, γ (°) | 90, 96.163 (8), 90 | 77.92 (2), 78.636 (18), 84.702 (17) |
| *V* (Å3) | 6398.3 (19) | 2888.6 (16) |
| *Z* | 4 | 2 |
| Radiation type | Mo *K*α | |
|  (mm-1) | 1.77 | 1.17 |
| Crystal size (mm) | 0.21 × 0.15 × 0.06 | 0.14 × 0.09 × 0.07 |
|  | | |
| Data collection | | |
| Diffractometer | Bruker D8 VENTURE | |
| Absorption correction | Multi-scan  *SADABS2016*/2 - Bruker AXS area detector scaling and absorption correction | |
| *T*min, *T*max | 0.71, 0.91 | 0.61, 0.93 |
| No. of measured, independent and  observed [*I* > 2σ(*I*)] reflections | 37401, 11506, 6916 | 22330, 7099, 3633 |
| *R*int | 0.185 | 0.177 |
| max (°) | 19.9 | 22.1 |
| (sin /λ)max (Å-1) | 0.479 | 0.530 |
|  | | |
| Refinement | | |
| *R*[*F*2 > 2σ(*F*2)], *wR*(*F*2), *S* | 0.075, 0.200, 1.00 | 0.089, 0.263, 1.02 |
| No. of reflections | 11506 | 7099 |
| No. of parameters | 1585 | 742 |
| No. of restraints | 1426 | 692 |
| H-atom treatment | H atoms treated by a mixture of independent and constrained refinement | H-atom parameters constrained |
| max, min (e Å-3) | 0.77, -0.56 | 0.86, -1.40 |
| Absolute structure | Refined as an inversion twin. | – |
| Absolute structure parameter | 0.43 (3) | – |

Table S3 Selected distance (Å), angle data for PdX(P-P(O)) moieties

|  | **2** | | **3** | WIGDOJ |
| --- | --- | --- | --- | --- |
| Pd-X (Å) | 2.460(5) | 2.447(5) | 2.366(3) | 2.270  2.341 |
| Pd-C (Å) | 1.96(3) | 1.99(3) | 1.940(13) | - |
| Pd-P (Å) | 2.258 (10) | 2.294 (10) | 2.265(3) | 2.235 |
| Pd-O (Å) | 2.151(19) | 2.20(2) | 2.153(9) | 2.083 |
| O-Pd-P (°) | 94.6(6) | 94.2(6) | 93.2(3) | 92.4 |
| P-Pd-X (°) | 175.6(3) | 174.6(3) | 176.78(14) | 175.3 |
| O-Pd-X (°) | 89.5(6) | 90.4(6) | 89.3(2) | 91.4 |
| C-Pd-X-P(O) | 165 | -168 | +/- 161 | - |
| Reference | This work | | This work | Ding et al. (Ref. [21] |

Table S4. Geometric parameters (Å, º) for **DPP-Bn**

| Distances (*Å)* | | | |
| --- | --- | --- | --- |
| C1—N1 | 1.363 (10) | C21—C26 | 1.402 (12) |
| C1—C20 | 1.383 (11) | C22—C23 | 1.391 (13) |
| C1—C2 | 1.435 (12) | C22—H22 | 0.9500 |
| C2—C3 | 1.333 (12) | C23—C24 | 1.369 (13) |
| C2—H2 | 0.9500 | C23—H23 | 0.9500 |
| C3—C4 | 1.428 (13) | C24—C25 | 1.383 (14) |
| C3—H3A | 0.9500 | C24—H24 | 0.9500 |
| C4—N1 | 1.350 (11) | C25—C26 | 1.374 (14) |
| C4—C5 | 1.406 (13) | C25—H25 | 0.9500 |
| C5—C6 | 1.374 (12) | C26—H26 | 0.9500 |
| C5—C50 | 1.489 (12) | C31—C36 | 1.365 (14) |
| C6—N2 | 1.370 (11) | C31—C32 | 1.369 (12) |
| C6—C7 | 1.413 (12) | C32—C33 | 1.374 (13) |
| C7—C8 | 1.356 (12) | C32—H32 | 0.9500 |
| C7—H7 | 0.9500 | C33—C34 | 1.348 (14) |
| C8—C9 | 1.441 (12) | C33—H33 | 0.9500 |
| C8—H8 | 0.9500 | C34—C35 | 1.340 (14) |
| C9—N2 | 1.338 (11) | C34—H34 | 0.9500 |
| C9—C10 | 1.389 (12) | C35—C36 | 1.382 (14) |
| C10—C11 | 1.406 (12) | C35—H35 | 0.9500 |
| C10—C21 | 1.474 (13) | C36—H36 | 0.9500 |
| C11—N3 | 1.361 (11) | C50—O50 | 1.213 (11) |
| C11—C12 | 1.435 (12) | C50—N50 | 1.334 (11) |
| C12—C13 | 1.338 (12) | C51—C52 | 1.370 (13) |
| C12—H12 | 0.9500 | C51—C56 | 1.387 (12) |
| C13—C14 | 1.414 (12) | C51—C57 | 1.494 (12) |
| C13—H13 | 0.9500 | C52—C53 | 1.376 (14) |
| C14—N3 | 1.379 (10) | C52—H52 | 0.9500 |
| C14—C15 | 1.386 (13) | C53—C54 | 1.343 (14) |
| C15—C16 | 1.351 (12) | C53—H53 | 0.9500 |
| C15—H15 | 0.9500 | C54—C55 | 1.374 (15) |
| C16—N4 | 1.341 (10) | C54—H54 | 0.9500 |
| C16—C17 | 1.428 (12) | C55—C56 | 1.370 (15) |
| C17—C18 | 1.330 (12) | C55—H55 | 0.9500 |
| C17—H17 | 0.9500 | C56—H56 | 0.9500 |
| C18—C19 | 1.422 (12) | C57—N50 | 1.435 (10) |
| C18—H18 | 0.9500 | C57—H57A | 0.9900 |
| C19—N4 | 1.362 (11) | C57—H57B | 0.9900 |
| C19—C20 | 1.385 (12) | N2—H2A | 0.8800 |
| C20—C31 | 1.504 (13) | N4—H4 | 0.8800 |
| C21—C22 | 1.358 (13) | N50—H50 | 0.89 (2) |
| Angles (º) | | | |
| N1—C1—C20 | 125.2 (8) | C24—C23—H23 | 119.9000 |
| N1—C1—C2 | 109.6 (7) | C22—C23—H23 | 119.9000 |
| C20—C1—C2 | 125.2 (8) | C23—C24—C25 | 118.4 (10) |
| C3—C2—C1 | 107.0 (8) | C23—C24—H24 | 120.8000 |
| C3—C2—H2 | 126.5000 | C25—C24—H24 | 120.8000 |
| C1—C2—H2 | 126.5000 | C26—C25—C24 | 121.2 (9) |
| C2—C3—C4 | 106.9 (8) | C26—C25—H25 | 119.4000 |
| C2—C3—H3A | 126.5000 | C24—C25—H25 | 119.4000 |
| C4—C3—H3A | 126.5000 | C25—C26—C21 | 120.6 (10) |
| N1—C4—C5 | 128.0 (8) | C25—C26—H26 | 119.7000 |
| N1—C4—C3 | 110.4 (8) | C21—C26—H26 | 119.7000 |
| C5—C4—C3 | 121.5 (9) | C36—C31—C32 | 118.3 (9) |
| C6—C5—C4 | 125.3 (8) | C36—C31—C20 | 122.5 (8) |
| C6—C5—C50 | 114.9 (8) | C32—C31—C20 | 119.2 (9) |
| C4—C5—C50 | 119.4 (9) | C31—C32—C33 | 119.4 (10) |
| N2—C6—C5 | 127.6 (8) | C31—C32—H32 | 120.3000 |
| N2—C6—C7 | 105.5 (9) | C33—C32—H32 | 120.3000 |
| C5—C6—C7 | 126.6 (9) | C34—C33—C32 | 121.9 (9) |
| C8—C7—C6 | 109.4 (8) | C34—C33—H33 | 119.1000 |
| C8—C7—H7 | 125.3000 | C32—C33—H33 | 119.1000 |
| C6—C7—H7 | 125.3000 | C35—C34—C33 | 119.2 (9) |
| C7—C8—C9 | 106.7 (8) | C35—C34—H34 | 120.4000 |
| C7—C8—H8 | 126.6000 | C33—C34—H34 | 120.4000 |
| C9—C8—H8 | 126.6000 | C34—C35—C36 | 120.1 (11) |
| N2—C9—C10 | 125.9 (8) | C34—C35—H35 | 120.0000 |
| N2—C9—C8 | 106.7 (8) | C36—C35—H35 | 120.0000 |
| C10—C9—C8 | 127.3 (9) | C31—C36—C35 | 121.1 (10) |
| C9—C10—C11 | 123.3 (9) | C31—C36—H36 | 119.5000 |
| C9—C10—C21 | 118.4 (8) | C35—C36—H36 | 119.5000 |
| C11—C10—C21 | 118.3 (8) | O50—C50—N50 | 123.5 (9) |
| N3—C11—C10 | 124.8 (8) | O50—C50—C5 | 120.7 (9) |
| N3—C11—C12 | 111.2 (7) | N50—C50—C5 | 115.8 (9) |
| C10—C11—C12 | 123.8 (9) | C52—C51—C56 | 117.1 (10) |
| C13—C12—C11 | 105.9 (9) | C52—C51—C57 | 121.2 (9) |
| C13—C12—H12 | 127.0000 | C56—C51—C57 | 121.7 (10) |
| C11—C12—H12 | 127.0000 | C51—C52—C53 | 121.3 (10) |
| C12—C13—C14 | 107.9 (8) | C51—C52—H52 | 119.3000 |
| C12—C13—H13 | 126.1000 | C53—C52—H52 | 119.3000 |
| C14—C13—H13 | 126.1000 | C54—C53—C52 | 120.8 (11) |
| N3—C14—C15 | 123.8 (9) | C54—C53—H53 | 119.6000 |
| N3—C14—C13 | 110.5 (8) | C52—C53—H53 | 119.6000 |
| C15—C14—C13 | 125.6 (9) | C53—C54—C55 | 119.4 (11) |
| C16—C15—C14 | 128.3 (9) | C53—C54—H54 | 120.3000 |
| C16—C15—H15 | 115.8000 | C55—C54—H54 | 120.3000 |
| C14—C15—H15 | 115.8000 | C56—C55—C54 | 120.1 (10) |
| N4—C16—C15 | 128.5 (8) | C56—C55—H55 | 120.0000 |
| N4—C16—C17 | 105.9 (8) | C54—C55—H55 | 120.0000 |
| C15—C16—C17 | 125.5 (9) | C55—C56—C51 | 121.2 (11) |
| C18—C17—C16 | 107.7 (8) | C55—C56—H56 | 119.4000 |
| C18—C17—H17 | 126.1000 | C51—C56—H56 | 119.4000 |
| C16—C17—H17 | 126.1000 | N50—C57—C51 | 114.0 (7) |
| C17—C18—C19 | 109.6 (8) | N50—C57—H57A | 108.8000 |
| C17—C18—H18 | 125.2000 | C51—C57—H57A | 108.8000 |
| C19—C18—H18 | 125.2000 | N50—C57—H57B | 108.8000 |
| N4—C19—C20 | 125.5 (8) | C51—C57—H57B | 108.8000 |
| N4—C19—C18 | 104.5 (7) | H57A—C57—H57B | 107.7000 |
| C20—C19—C18 | 129.9 (9) | C4—N1—C1 | 106.1 (7) |
| C19—C20—C1 | 124.3 (8) | C9—N2—C6 | 111.6 (7) |
| C19—C20—C31 | 115.7 (8) | C9—N2—H2A | 124.2000 |
| C1—C20—C31 | 120.1 (8) | C6—N2—H2A | 124.2000 |
| C22—C21—C26 | 117.4 (10) | C11—N3—C14 | 104.5 (7) |
| C22—C21—C10 | 120.0 (8) | C16—N4—C19 | 112.1 (7) |
| C26—C21—C10 | 122.5 (10) | C16—N4—H4 | 123.9000 |
| C21—C22—C23 | 122.1 (8) | C19—N4—H4 | 123.9000 |
| C21—C22—H22 | 118.9000 | C50—N50—C57 | 122.7 (7) |
| C23—C22—H22 | 118.9000 | C50—N50—H50 | 117 (5) |
| C24—C23—C22 | 120.2 (10) | C57—N50—H50 | 120 (5) |
| Torsion angles (º) | | | |
| N1—C1—C2—C3 | -2.5 (10) | C10—C21—C22—C23 | -179.9 (8) |
| C20—C1—C2—C3 | 173.7 (8) | C21—C22—C23—C24 | 1.7 (14) |
| C1—C2—C3—C4 | 1.8 (10) | C22—C23—C24—C25 | -0.5 (14) |
| C2—C3—C4—N1 | -0.5 (10) | C23—C24—C25—C26 | 0.7 (15) |
| C2—C3—C4—C5 | -179.4 (8) | C24—C25—C26—C21 | -2.1 (15) |
| N1—C4—C5—C6 | 5.6 (15) | C22—C21—C26—C25 | 3.1 (13) |
| C3—C4—C5—C6 | -175.7 (9) | C10—C21—C26—C25 | -180.0 (9) |
| N1—C4—C5—C50 | -166.4 (9) | C19—C20—C31—C36 | 102.6 (11) |
| C3—C4—C5—C50 | 12.3 (13) | C1—C20—C31—C36 | -76.7 (12) |
| C4—C5—C6—N2 | -3.4 (15) | C19—C20—C31—C32 | -77.2 (10) |
| C50—C5—C6—N2 | 169.0 (9) | C1—C20—C31—C32 | 103.5 (10) |
| C4—C5—C6—C7 | 169.7 (9) | C36—C31—C32—C33 | -1.9 (14) |
| C50—C5—C6—C7 | -17.9 (13) | C20—C31—C32—C33 | 177.9 (8) |
| N2—C6—C7—C8 | 1.0 (10) | C31—C32—C33—C34 | -0.9 (14) |
| C5—C6—C7—C8 | -173.4 (9) | C32—C33—C34—C35 | 3.0 (16) |
| C6—C7—C8—C9 | -1.7 (10) | C33—C34—C35—C36 | -2.0 (17) |
| C7—C8—C9—N2 | 1.9 (10) | C32—C31—C36—C35 | 2.8 (16) |
| C7—C8—C9—C10 | -176.5 (9) | C20—C31—C36—C35 | -177.0 (10) |
| N2—C9—C10—C11 | -8.0 (14) | C34—C35—C36—C31 | -0.9 (19) |
| C8—C9—C10—C11 | 170.1 (8) | C6—C5—C50—O50 | -73.0 (12) |
| N2—C9—C10—C21 | 172.3 (8) | C4—C5—C50—O50 | 99.9 (10) |
| C8—C9—C10—C21 | -9.6 (13) | C6—C5—C50—N50 | 108.4 (9) |
| C9—C10—C11—N3 | 4.0 (14) | C4—C5—C50—N50 | -78.7 (11) |
| C21—C10—C11—N3 | -176.2 (8) | C56—C51—C52—C53 | 3.9 (13) |
| C9—C10—C11—C12 | -170.3 (8) | C57—C51—C52—C53 | -177.1 (8) |
| C21—C10—C11—C12 | 9.5 (13) | C51—C52—C53—C54 | -1.5 (14) |
| N3—C11—C12—C13 | -1.9 (10) | C52—C53—C54—C55 | -1.4 (15) |
| C10—C11—C12—C13 | 173.1 (8) | C53—C54—C55—C56 | 1.7 (15) |
| C11—C12—C13—C14 | 2.5 (10) | C54—C55—C56—C51 | 0.9 (14) |
| C12—C13—C14—N3 | -2.4 (10) | C52—C51—C56—C55 | -3.6 (12) |
| C12—C13—C14—C15 | 173.3 (9) | C57—C51—C56—C55 | 177.5 (8) |
| N3—C14—C15—C16 | 5.3 (15) | C52—C51—C57—N50 | 57.2 (10) |
| C13—C14—C15—C16 | -170.0 (9) | C56—C51—C57—N50 | -124.0 (8) |
| C14—C15—C16—N4 | -6.7 (16) | C5—C4—N1—C1 | 177.8 (9) |
| C14—C15—C16—C17 | 171.0 (9) | C3—C4—N1—C1 | -1.0 (9) |
| N4—C16—C17—C18 | 2.4 (11) | C20—C1—N1—C4 | -174.1 (8) |
| C15—C16—C17—C18 | -175.7 (9) | C2—C1—N1—C4 | 2.1 (9) |
| C16—C17—C18—C19 | -1.5 (11) | C10—C9—N2—C6 | 177.1 (8) |
| C17—C18—C19—N4 | 0.1 (10) | C8—C9—N2—C6 | -1.3 (10) |
| C17—C18—C19—C20 | -177.4 (9) | C5—C6—N2—C9 | 174.6 (9) |
| N4—C19—C20—C1 | -3.7 (14) | C7—C6—N2—C9 | 0.3 (10) |
| C18—C19—C20—C1 | 173.3 (9) | C10—C11—N3—C14 | -174.5 (8) |
| N4—C19—C20—C31 | 177.1 (8) | C12—C11—N3—C14 | 0.4 (9) |
| C18—C19—C20—C31 | -5.9 (13) | C15—C14—N3—C11 | -174.7 (9) |
| N1—C1—C20—C19 | 7.7 (14) | C13—C14—N3—C11 | 1.2 (9) |
| C2—C1—C20—C19 | -168.0 (8) | C15—C16—N4—C19 | 175.6 (9) |
| N1—C1—C20—C31 | -173.1 (8) | C17—C16—N4—C19 | -2.5 (10) |
| C2—C1—C20—C31 | 11.2 (13) | C20—C19—N4—C16 | 179.2 (8) |
| C9—C10—C21—C22 | -117.4 (9) | C18—C19—N4—C16 | 1.5 (9) |
| C11—C10—C21—C22 | 62.9 (11) | O50—C50—N50—C57 | -0.4 (14) |
| C9—C10—C21—C26 | 65.8 (11) | C5—C50—N50—C57 | 178.1 (8) |
| C11—C10—C21—C26 | -114.0 (10) | C51—C57—N50—C50 | -87.7 (10) |
| C26—C21—C22—C23 | -3.0 (13) |  |  |

Table S5. Geometric parameters (Å, º) for **DPP-Gly**

| Distances (*Å)* | | | |
| --- | --- | --- | --- |
| C1—N1 | 1.363 (12) | C21—C22 | 1.375 (13) |
| C1—C20 | 1.381 (13) | C22—C23 | 1.348 (15) |
| C1—C2 | 1.434 (11) | C22—H22 | 0.9300 |
| C2—C3 | 1.327 (13) | C23—C24 | 1.363 (18) |
| C2—H2 | 0.9300 | C23—H23 | 0.9300 |
| C3—C4 | 1.436 (13) | C24—C25 | 1.335 (17) |
| C3—H3 | 0.9300 | C24—H24 | 0.9300 |
| C4—N1 | 1.347 (12) | C25—C26 | 1.389 (16) |
| C4—C5 | 1.373 (14) | C25—H25 | 0.9300 |
| C5—C6 | 1.411 (13) | C26—H26 | 0.9300 |
| C5—H5 | 0.9300 | C31—C32 | 1.359 (13) |
| C6—N2 | 1.363 (11) | C31—C36 | 1.379 (13) |
| C6—C7 | 1.430 (13) | C32—C33 | 1.392 (14) |
| C7—C8 | 1.333 (12) | C32—H32 | 0.9300 |
| C7—H7 | 0.9300 | C33—C34 | 1.353 (17) |
| C8—C9 | 1.421 (13) | C33—H33 | 0.9300 |
| C8—H8 | 0.9300 | C34—C35 | 1.335 (17) |
| C9—N2 | 1.388 (11) | C34—H34 | 0.9300 |
| C9—C10 | 1.400 (12) | C35—C36 | 1.428 (16) |
| C10—C11 | 1.387 (12) | C35—H35 | 0.9300 |
| C10—C21 | 1.497 (13) | C36—H36 | 0.9300 |
| C11—N3 | 1.357 (11) | C51—O51 | 1.215 (10) |
| C11—C12 | 1.411 (11) | C51—N51 | 1.333 (11) |
| C12—C13 | 1.322 (12) | C52—N51 | 1.451 (10) |
| C12—H12 | 0.9300 | C52—C53 | 1.496 (13) |
| C13—C14 | 1.453 (11) | C52—H52A | 0.9700 |
| C13—H13 | 0.9300 | C52—H52B | 0.9700 |
| C14—N3 | 1.347 (11) | C53—O53 | 1.193 (10) |
| C14—C15 | 1.376 (12) | C53—O54 | 1.320 (10) |
| C15—C16 | 1.407 (11) | C54—O54 | 1.430 (11) |
| C15—C51 | 1.479 (11) | C54—H54A | 0.9600 |
| C16—N4 | 1.363 (10) | C54—H54B | 0.9600 |
| C16—C17 | 1.427 (12) | C54—H54C | 0.9600 |
| C17—C18 | 1.375 (11) | C90—Cl1 | 1.731 (12) |
| C17—H17 | 0.9300 | C90—Cl3 | 1.736 (12) |
| C18—C19 | 1.415 (12) | C90—Cl2 | 1.737 (12) |
| C18—H18 | 0.9300 | C90—H90 | 0.9800 |
| C19—N4 | 1.373 (11) | N1—H1N | 0.86 (2) |
| C19—C20 | 1.418 (11) | N3—H3N | 0.86 (2) |
| C20—C31 | 1.503 (12) | N51—H51N | 0.87 (2) |
| C21—C26 | 1.359 (14) |  |  |
| Angles (º) | | | |
| N1—C1—C20 | 125.3 (8) | C22—C23—C24 | 120.4 (13) |
| N1—C1—C2 | 107.2 (9) | C22—C23—H23 | 119.8000 |
| C20—C1—C2 | 127.5 (10) | C24—C23—H23 | 119.8000 |
| C3—C2—C1 | 107.4 (9) | C25—C24—C23 | 119.5 (13) |
| C3—C2—H2 | 126.3000 | C25—C24—H24 | 120.3000 |
| C1—C2—H2 | 126.3000 | C23—C24—H24 | 120.3000 |
| C2—C3—C4 | 108.7 (9) | C24—C25—C26 | 120.0 (13) |
| C2—C3—H3 | 125.7000 | C24—C25—H25 | 120.0000 |
| C4—C3—H3 | 125.7000 | C26—C25—H25 | 120.0000 |
| N1—C4—C5 | 126.9 (9) | C21—C26—C25 | 121.3 (12) |
| N1—C4—C3 | 106.9 (10) | C21—C26—H26 | 119.3000 |
| C5—C4—C3 | 125.7 (9) | C25—C26—H26 | 119.3000 |
| C4—C5—C6 | 129.8 (9) | C32—C31—C36 | 120.9 (9) |
| C4—C5—H5 | 115.1000 | C32—C31—C20 | 121.6 (9) |
| C6—C5—H5 | 115.1000 | C36—C31—C20 | 117.2 (9) |
| N2—C6—C5 | 123.7 (10) | C31—C32—C33 | 120.6 (10) |
| N2—C6—C7 | 110.6 (8) | C31—C32—H32 | 119.7000 |
| C5—C6—C7 | 125.6 (9) | C33—C32—H32 | 119.7000 |
| C8—C7—C6 | 106.8 (8) | C34—C33—C32 | 119.7 (12) |
| C8—C7—H7 | 126.6000 | C34—C33—H33 | 120.2000 |
| C6—C7—H7 | 126.6000 | C32—C33—H33 | 120.2000 |
| C7—C8—C9 | 107.9 (9) | C35—C34—C33 | 120.2 (12) |
| C7—C8—H8 | 126.0000 | C35—C34—H34 | 119.9000 |
| C9—C8—H8 | 126.0000 | C33—C34—H34 | 119.9000 |
| N2—C9—C10 | 122.9 (9) | C34—C35—C36 | 122.0 (12) |
| N2—C9—C8 | 109.6 (8) | C34—C35—H35 | 119.0000 |
| C10—C9—C8 | 127.4 (10) | C36—C35—H35 | 119.0000 |
| C11—C10—C9 | 124.7 (9) | C31—C36—C35 | 116.5 (11) |
| C11—C10—C21 | 117.2 (8) | C31—C36—H36 | 121.7000 |
| C9—C10—C21 | 118.0 (9) | C35—C36—H36 | 121.7000 |
| N3—C11—C10 | 125.4 (8) | O51—C51—N51 | 123.2 (8) |
| N3—C11—C12 | 107.1 (8) | O51—C51—C15 | 121.4 (8) |
| C10—C11—C12 | 127.5 (9) | N51—C51—C15 | 115.3 (8) |
| C13—C12—C11 | 109.1 (8) | N51—C52—C53 | 114.2 (7) |
| C13—C12—H12 | 125.5000 | N51—C52—H52A | 108.7000 |
| C11—C12—H12 | 125.5000 | C53—C52—H52A | 108.7000 |
| C12—C13—C14 | 107.4 (8) | N51—C52—H52B | 108.7000 |
| C12—C13—H13 | 126.3000 | C53—C52—H52B | 108.7000 |
| C14—C13—H13 | 126.3000 | H52A—C52—H52B | 107.6000 |
| N3—C14—C15 | 128.6 (8) | O53—C53—O54 | 125.3 (10) |
| N3—C14—C13 | 106.3 (8) | O53—C53—C52 | 126.2 (9) |
| C15—C14—C13 | 125.0 (8) | O54—C53—C52 | 108.4 (8) |
| C14—C15—C16 | 126.3 (8) | O54—C54—H54A | 109.5000 |
| C14—C15—C51 | 117.8 (7) | O54—C54—H54B | 109.5000 |
| C16—C15—C51 | 115.8 (8) | H54A—C54—H54B | 109.5000 |
| N4—C16—C15 | 126.1 (8) | O54—C54—H54C | 109.5000 |
| N4—C16—C17 | 111.5 (7) | H54A—C54—H54C | 109.5000 |
| C15—C16—C17 | 122.4 (8) | H54B—C54—H54C | 109.5000 |
| C18—C17—C16 | 106.2 (8) | Cl1—C90—Cl3 | 109.6 (6) |
| C18—C17—H17 | 126.9000 | Cl1—C90—Cl2 | 110.1 (7) |
| C16—C17—H17 | 126.9000 | Cl3—C90—Cl2 | 110.4 (7) |
| C17—C18—C19 | 105.9 (9) | Cl1—C90—H90 | 108.9000 |
| C17—C18—H18 | 127.1000 | Cl3—C90—H90 | 108.9000 |
| C19—C18—H18 | 127.1000 | Cl2—C90—H90 | 108.9000 |
| N4—C19—C18 | 112.0 (7) | C4—N1—C1 | 109.7 (8) |
| N4—C19—C20 | 122.4 (9) | C4—N1—H1N | 136 (9) |
| C18—C19—C20 | 125.6 (9) | C1—N1—H1N | 115 (9) |
| C1—C20—C19 | 124.5 (9) | C6—N2—C9 | 105.0 (8) |
| C1—C20—C31 | 118.3 (8) | C14—N3—C11 | 110.2 (7) |
| C19—C20—C31 | 116.9 (8) | C14—N3—H3N | 123 (9) |
| C26—C21—C22 | 116.9 (10) | C11—N3—H3N | 126 (9) |
| C26—C21—C10 | 120.9 (10) | C16—N4—C19 | 104.4 (7) |
| C22—C21—C10 | 122.2 (10) | C51—N51—C52 | 120.8 (7) |
| C23—C22—C21 | 121.7 (11) | C51—N51—H51N | 117 (9) |
| C23—C22—H22 | 119.1000 | C52—N51—H51N | 122 (9) |
| C21—C22—H22 | 119.1000 | C53—O54—C54 | 117.5 (8) |
| Torsion angles (º) | | | |
| N1—C1—C2—C3 | 1.8 (10) | C11—C10—C21—C22 | -120.4 (10) |
| C20—C1—C2—C3 | -178.8 (9) | C9—C10—C21—C22 | 57.0 (12) |
| C1—C2—C3—C4 | -0.7 (10) | C26—C21—C22—C23 | -2.1 (15) |
| C2—C3—C4—N1 | -0.6 (10) | C10—C21—C22—C23 | 177.0 (10) |
| C2—C3—C4—C5 | 171.4 (9) | C21—C22—C23—C24 | 4.3 (18) |
| N1—C4—C5—C6 | 1.2 (16) | C22—C23—C24—C25 | -5 (2) |
| C3—C4—C5—C6 | -169.2 (9) | C23—C24—C25—C26 | 3 (2) |
| C4—C5—C6—N2 | -7.2 (15) | C22—C21—C26—C25 | 0.5 (15) |
| C4—C5—C6—C7 | 170.0 (9) | C10—C21—C26—C25 | -178.6 (10) |
| N2—C6—C7—C8 | 0.6 (10) | C24—C25—C26—C21 | -1.2 (19) |
| C5—C6—C7—C8 | -176.9 (8) | C1—C20—C31—C32 | -121.4 (10) |
| C6—C7—C8—C9 | -1.4 (10) | C19—C20—C31—C32 | 64.5 (12) |
| C7—C8—C9—N2 | 1.8 (10) | C1—C20—C31—C36 | 64.8 (11) |
| C7—C8—C9—C10 | -176.4 (8) | C19—C20—C31—C36 | -109.3 (9) |
| N2—C9—C10—C11 | 1.8 (13) | C36—C31—C32—C33 | 3.5 (15) |
| C8—C9—C10—C11 | 179.7 (9) | C20—C31—C32—C33 | -170.0 (9) |
| N2—C9—C10—C21 | -175.4 (8) | C31—C32—C33—C34 | -2.0 (16) |
| C8—C9—C10—C21 | 2.6 (13) | C32—C33—C34—C35 | -0.5 (18) |
| C9—C10—C11—N3 | 6.4 (14) | C33—C34—C35—C36 | 1.5 (19) |
| C21—C10—C11—N3 | -176.4 (8) | C32—C31—C36—C35 | -2.5 (13) |
| C9—C10—C11—C12 | -171.5 (8) | C20—C31—C36—C35 | 171.3 (8) |
| C21—C10—C11—C12 | 5.7 (13) | C34—C35—C36—C31 | 0.0 (16) |
| N3—C11—C12—C13 | 0.4 (10) | C14—C15—C51—O51 | 87.5 (11) |
| C10—C11—C12—C13 | 178.6 (8) | C16—C15—C51—O51 | -89.3 (10) |
| C11—C12—C13—C14 | -0.6 (9) | C14—C15—C51—N51 | -88.4 (9) |
| C12—C13—C14—N3 | 0.5 (9) | C16—C15—C51—N51 | 94.8 (9) |
| C12—C13—C14—C15 | 176.3 (8) | N51—C52—C53—O53 | -9.0 (13) |
| N3—C14—C15—C16 | 1.0 (14) | N51—C52—C53—O54 | 172.1 (7) |
| C13—C14—C15—C16 | -173.9 (8) | C5—C4—N1—C1 | -170.1 (9) |
| N3—C14—C15—C51 | -175.5 (8) | C3—C4—N1—C1 | 1.8 (10) |
| C13—C14—C15—C51 | 9.6 (12) | C20—C1—N1—C4 | 178.4 (8) |
| C14—C15—C16—N4 | -7.9 (14) | C2—C1—N1—C4 | -2.2 (10) |
| C51—C15—C16—N4 | 168.7 (8) | C5—C6—N2—C9 | 178.0 (8) |
| C14—C15—C16—C17 | 172.0 (8) | C7—C6—N2—C9 | 0.5 (9) |
| C51—C15—C16—C17 | -11.5 (12) | C10—C9—N2—C6 | 176.9 (8) |
| N4—C16—C17—C18 | 1.2 (10) | C8—C9—N2—C6 | -1.4 (9) |
| C15—C16—C17—C18 | -178.7 (8) | C15—C14—N3—C11 | -175.9 (8) |
| C16—C17—C18—C19 | -2.1 (10) | C13—C14—N3—C11 | -0.2 (9) |
| C17—C18—C19—N4 | 2.5 (10) | C10—C11—N3—C14 | -178.4 (8) |
| C17—C18—C19—C20 | -175.6 (8) | C12—C11—N3—C14 | -0.1 (9) |
| N1—C1—C20—C19 | 4.0 (14) | C15—C16—N4—C19 | -179.9 (8) |
| C2—C1—C20—C19 | -175.3 (8) | C17—C16—N4—C19 | 0.3 (9) |
| N1—C1—C20—C31 | -169.7 (8) | C18—C19—N4—C16 | -1.7 (9) |
| C2—C1—C20—C31 | 11.0 (14) | C20—C19—N4—C16 | 176.4 (7) |
| N4—C19—C20—C1 | -3.5 (13) | O51—C51—N51—C52 | 0.1 (14) |
| C18—C19—C20—C1 | 174.3 (9) | C15—C51—N51—C52 | 175.9 (7) |
| N4—C19—C20—C31 | 170.2 (8) | C53—C52—N51—C51 | 83.6 (10) |
| C18—C19—C20—C31 | -12.0 (13) | O53—C53—O54—C54 | -1.4 (13) |
| C11—C10—C21—C26 | 58.6 (12) | C52—C53—O54—C54 | 177.6 (8) |
| C9—C10—C21—C26 | -124.0 (10) |  |  |

Table S6. Geometric parameters (Å, º) for **DPP-Gly2**

| Distances (*Å)* | | | |
| --- | --- | --- | --- |
| C1—N1 | 1.372 (4) | C23—H23 | 0.9300 |
| C1—C20 | 1.398 (5) | C24—C25 | 1.364 (6) |
| C1—C2 | 1.429 (5) | C24—H24 | 0.9300 |
| C2—C3 | 1.349 (5) | C25—C26 | 1.388 (5) |
| C2—H2 | 0.9300 | C25—H25 | 0.9300 |
| C3—C4 | 1.425 (4) | C26—H26 | 0.9300 |
| C3—H3 | 0.9300 | C40—O40 | 1.206 (4) |
| C4—N1 | 1.370 (4) | C40—N40 | 1.311 (5) |
| C4—C5 | 1.393 (4) | C41—O42 | 1.180 (5) |
| C5—C6 | 1.397 (4) | C41—O41 | 1.321 (5) |
| C5—C40 | 1.514 (4) | C41—C42 | 1.496 (6) |
| C6—N4 | 1.371 (4) | C42—N40 | 1.452 (5) |
| C6—C7 | 1.435 (5) | C42—H42A | 0.9700 |
| C7—C8 | 1.346 (5) | C42—H42B | 0.9700 |
| C7—H7 | 0.9300 | C43—O41 | 1.460 (6) |
| C8—C19i | 1.433 (5) | C43—H43A | 0.9600 |
| C8—H8 | 0.9300 | C43—H43B | 0.9600 |
| C19—N4i | 1.379 (4) | C43—H43C | 0.9600 |
| C19—C20 | 1.396 (5) | C90—Cl2 | 1.725 (5) |
| C20—C21 | 1.496 (4) | C90—Cl3 | 1.733 (5) |
| C21—C22 | 1.380 (5) | C90—Cl1 | 1.736 (5) |
| C21—C26 | 1.382 (5) | C90—H90 | 0.9800 |
| C22—C23 | 1.391 (5) | N1—H1 | 0.856 (19) |
| C22—H22 | 0.9300 | N40—H40 | 0.854 (19) |
| C23—C24 | 1.359 (7) |  |  |
| Angles (º) | | | |
| N1—C1—C20 | 126.2 (3) | C23—C24—H24 | 120.1000 |
| N1—C1—C2 | 107.8 (3) | C25—C24—H24 | 120.1000 |
| C20—C1—C2 | 126.0 (3) | C24—C25—C26 | 120.8 (4) |
| C3—C2—C1 | 107.9 (3) | C24—C25—H25 | 119.6000 |
| C3—C2—H2 | 126.1000 | C26—C25—H25 | 119.6000 |
| C1—C2—H2 | 126.1000 | C21—C26—C25 | 120.0 (4) |
| C2—C3—C4 | 107.8 (3) | C21—C26—H26 | 120.0000 |
| C2—C3—H3 | 126.1000 | C25—C26—H26 | 120.0000 |
| C4—C3—H3 | 126.1000 | O40—C40—N40 | 122.0 (3) |
| N1—C4—C5 | 126.2 (3) | O40—C40—C5 | 120.8 (3) |
| N1—C4—C3 | 108.1 (3) | N40—C40—C5 | 117.2 (3) |
| C5—C4—C3 | 125.7 (3) | O42—C41—O41 | 124.2 (4) |
| C4—C5—C6 | 126.9 (3) | O42—C41—C42 | 125.6 (4) |
| C4—C5—C40 | 115.5 (3) | O41—C41—C42 | 110.2 (4) |
| C6—C5—C40 | 117.4 (3) | N40—C42—C41 | 112.1 (4) |
| N4—C6—C5 | 125.1 (3) | N40—C42—H42A | 109.2000 |
| N4—C6—C7 | 109.3 (3) | C41—C42—H42A | 109.2000 |
| C5—C6—C7 | 125.6 (3) | N40—C42—H42B | 109.2000 |
| C8—C7—C6 | 107.5 (3) | C41—C42—H42B | 109.2000 |
| C8—C7—H7 | 126.2000 | H42A—C42—H42B | 107.9000 |
| C6—C7—H7 | 126.2000 | O41—C43—H43A | 109.5000 |
| C7—C8—C19i | 107.2 (3) | O41—C43—H43B | 109.5000 |
| C7—C8—H8 | 126.4000 | H43A—C43—H43B | 109.5000 |
| C19i—C8—H8 | 126.4000 | O41—C43—H43C | 109.5000 |
| N4i—C19—C20 | 125.6 (3) | H43A—C43—H43C | 109.5000 |
| N4i—C19—C8i | 109.4 (3) | H43B—C43—H43C | 109.5000 |
| C20—C19—C8i | 125.0 (3) | Cl2—C90—Cl3 | 110.2 (3) |
| C19—C20—C1 | 125.0 (3) | Cl2—C90—Cl1 | 113.1 (3) |
| C19—C20—C21 | 118.4 (3) | Cl3—C90—Cl1 | 109.1 (3) |
| C1—C20—C21 | 116.5 (3) | Cl2—C90—H90 | 108.1000 |
| C22—C21—C26 | 118.7 (3) | Cl3—C90—H90 | 108.1000 |
| C22—C21—C20 | 121.9 (3) | Cl1—C90—H90 | 108.1000 |
| C26—C21—C20 | 119.3 (3) | C4—N1—C1 | 108.4 (3) |
| C21—C22—C23 | 120.4 (4) | C4—N1—H1 | 126 (3) |
| C21—C22—H22 | 119.8000 | C1—N1—H1 | 126 (3) |
| C23—C22—H22 | 119.8000 | C6—N4—C19i | 106.5 (3) |
| C24—C23—C22 | 120.3 (4) | C40—N40—C42 | 121.2 (3) |
| C24—C23—H23 | 119.8000 | C40—N40—H40 | 119 (3) |
| C22—C23—H23 | 119.8000 | C42—N40—H40 | 119 (3) |
| C23—C24—C25 | 119.7 (4) | C41—O41—C43 | 116.2 (4) |
| Torsion angles (º) | | | |
| N1—C1—C2—C3 | 1.7 (4) | C1—C20—C21—C26 | 105.9 (4) |
| C20—C1—C2—C3 | -175.6 (3) | C26—C21—C22—C23 | -0.8 (6) |
| C1—C2—C3—C4 | -0.3 (4) | C20—C21—C22—C23 | 176.2 (3) |
| C2—C3—C4—N1 | -1.3 (4) | C21—C22—C23—C24 | -0.2 (6) |
| C2—C3—C4—C5 | 175.8 (3) | C22—C23—C24—C25 | 0.6 (7) |
| N1—C4—C5—C6 | -3.4 (6) | C23—C24—C25—C26 | 0.0 (7) |
| C3—C4—C5—C6 | 180.0 (3) | C22—C21—C26—C25 | 1.4 (5) |
| N1—C4—C5—C40 | 170.6 (3) | C20—C21—C26—C25 | -175.7 (3) |
| C3—C4—C5—C40 | -6.0 (5) | C24—C25—C26—C21 | -1.0 (6) |
| C4—C5—C6—N4 | 1.2 (5) | C4—C5—C40—O40 | -92.0 (4) |
| C40—C5—C6—N4 | -172.8 (3) | C6—C5—C40—O40 | 82.6 (4) |
| C4—C5—C6—C7 | -178.8 (3) | C4—C5—C40—N40 | 87.3 (4) |
| C40—C5—C6—C7 | 7.3 (5) | C6—C5—C40—N40 | -98.1 (4) |
| N4—C6—C7—C8 | -1.1 (4) | O42—C41—C42—N40 | -5.5 (6) |
| C5—C6—C7—C8 | 178.9 (3) | O41—C41—C42—N40 | 175.0 (3) |
| C6—C7—C8—C19i | 1.0 (4) | C5—C4—N1—C1 | -174.7 (3) |
| N4i—C19—C20—C1 | -3.7 (5) | C3—C4—N1—C1 | 2.4 (4) |
| C8i—C19—C20—C1 | 172.3 (3) | C20—C1—N1—C4 | 174.8 (3) |
| N4i—C19—C20—C21 | 172.4 (3) | C2—C1—N1—C4 | -2.5 (4) |
| C8i—C19—C20—C21 | -11.5 (5) | C5—C6—N4—C19i | -179.3 (3) |
| N1—C1—C20—C19 | 2.3 (5) | C7—C6—N4—C19i | 0.7 (4) |
| C2—C1—C20—C19 | 179.2 (3) | O40—C40—N40—C42 | 6.2 (6) |
| N1—C1—C20—C21 | -173.9 (3) | C5—C40—N40—C42 | -173.1 (3) |
| C2—C1—C20—C21 | 3.0 (5) | C41—C42—N40—C40 | 85.1 (5) |
| C19—C20—C21—C22 | 112.4 (4) | O42—C41—O41—C43 | -3.9 (7) |
| C1—C20—C21—C22 | -71.1 (4) | C42—C41—O41—C43 | 175.6 (4) |
| C19—C20—C21—C26 | -70.6 (4) |  |  |

Symmetry code: (i) -*x*, -*y*+1, -*z*+1.

Table S7. Geometric parameters (Å, º) for **DPP-Ala2**

| Distances (*Å)* | | | |
| --- | --- | --- | --- |
| C1—N1 | 1.377 (14) | C25—C26 | 1.399 (17) |
| C1—C20 | 1.382 (15) | C25—H25 | 0.9300 |
| C1—C2 | 1.417 (16) | C26—H26 | 0.9300 |
| C2—C3 | 1.343 (15) | C31—C32 | 1.298 (18) |
| C2—H2 | 0.9300 | C31—C36 | 1.312 (19) |
| C3—C4 | 1.411 (16) | C32—C33 | 1.42 (2) |
| C3—H3A | 0.9300 | C32—H32 | 0.9300 |
| C4—C5 | 1.376 (15) | C33—C34 | 1.30 (2) |
| C4—N1 | 1.378 (14) | C33—H33 | 0.9300 |
| C5—C6 | 1.397 (15) | C34—C35 | 1.29 (2) |
| C5—C50 | 1.478 (16) | C34—H34 | 0.9300 |
| C6—N2 | 1.365 (13) | C35—C36 | 1.38 (2) |
| C6—C7 | 1.442 (15) | C35—H35 | 0.9300 |
| C7—C8 | 1.332 (15) | C36—H36 | 0.9300 |
| C7—H7 | 0.9300 | C50—O50 | 1.228 (13) |
| C8—C9 | 1.425 (15) | C50—N50 | 1.327 (14) |
| C8—H8 | 0.9300 | C51—N50 | 1.463 (13) |
| C9—N2 | 1.378 (13) | C51—C54 | 1.504 (16) |
| C9—C10 | 1.411 (15) | C51—C52 | 1.526 (19) |
| C10—C11 | 1.394 (14) | C51—H51 | 0.9800 |
| C10—C21 | 1.480 (16) | C52—O51 | 1.164 (15) |
| C11—N3 | 1.378 (14) | C52—O52 | 1.280 (16) |
| C11—C12 | 1.420 (15) | C53—O52 | 1.457 (15) |
| C12—C13 | 1.359 (15) | C53—H53A | 0.9600 |
| C12—H12 | 0.9300 | C53—H53B | 0.9600 |
| C13—C14 | 1.411 (15) | C53—H53C | 0.9600 |
| C13—H13 | 0.9300 | C54—H54A | 0.9600 |
| C14—N3 | 1.378 (14) | C54—H54B | 0.9600 |
| C14—C15 | 1.378 (15) | C54—H54C | 0.9600 |
| C15—C16 | 1.383 (15) | C60—O60 | 1.232 (13) |
| C15—C60 | 1.510 (16) | C60—N60 | 1.316 (14) |
| C16—N4 | 1.374 (13) | C61—N60 | 1.457 (14) |
| C16—C17 | 1.441 (16) | C61—C62 | 1.466 (17) |
| C17—C18 | 1.314 (16) | C61—C64 | 1.528 (16) |
| C17—H17 | 0.9300 | C61—H61 | 0.9800 |
| C18—C19 | 1.447 (15) | C62—O61 | 1.184 (16) |
| C18—H18 | 0.9300 | C62—O62 | 1.314 (17) |
| C19—N4 | 1.374 (15) | C63—O62 | 1.452 (16) |
| C19—C20 | 1.400 (16) | C63—H63A | 0.9600 |
| C20—C31 | 1.499 (17) | C63—H63B | 0.9600 |
| C21—C26 | 1.379 (16) | C63—H63C | 0.9600 |
| C21—C22 | 1.384 (15) | C64—H64A | 0.9600 |
| C22—C23 | 1.377 (17) | C64—H64B | 0.9600 |
| C22—H22 | 0.9300 | C64—H64C | 0.9600 |
| C23—C24 | 1.362 (19) | N1—H1 | 0.8600 (16) |
| C23—H23 | 0.9300 | N3—H3 | 0.8600 (16) |
| C24—C25 | 1.322 (18) | N50—H50 | 0.8600 |
| C24—H24 | 0.9300 | N60—H60 | 0.8600 |
| Angles (º) | | | |
| N1—C1—C20 | 123.7 (13) | C36—C31—C20 | 122.8 (15) |
| N1—C1—C2 | 106.1 (12) | C31—C32—C33 | 120.7 (17) |
| C20—C1—C2 | 130.1 (14) | C31—C32—H32 | 119.7000 |
| C3—C2—C1 | 108.9 (11) | C33—C32—H32 | 119.7000 |
| C3—C2—H2 | 125.6000 | C34—C33—C32 | 122.2 (18) |
| C1—C2—H2 | 125.6000 | C34—C33—H33 | 118.9000 |
| C2—C3—C4 | 108.7 (11) | C32—C33—H33 | 118.9000 |
| C2—C3—H3A | 125.7000 | C35—C34—C33 | 116.7 (19) |
| C4—C3—H3A | 125.7000 | C35—C34—H34 | 121.6000 |
| C5—C4—N1 | 127.4 (12) | C33—C34—H34 | 121.6000 |
| C5—C4—C3 | 126.0 (13) | C34—C35—C36 | 120.8 (18) |
| N1—C4—C3 | 106.4 (11) | C34—C35—H35 | 119.6000 |
| C4—C5—C6 | 127.6 (11) | C36—C35—H35 | 119.6000 |
| C4—C5—C50 | 114.9 (12) | C31—C36—C35 | 123.8 (16) |
| C6—C5—C50 | 117.5 (11) | C31—C36—H36 | 118.1000 |
| N2—C6—C5 | 125.6 (12) | C35—C36—H36 | 118.1000 |
| N2—C6—C7 | 110.9 (12) | O50—C50—N50 | 123.5 (13) |
| C5—C6—C7 | 123.6 (13) | O50—C50—C5 | 121.8 (13) |
| C8—C7—C6 | 106.9 (11) | N50—C50—C5 | 114.7 (12) |
| C8—C7—H7 | 126.5000 | N50—C51—C54 | 111.3 (11) |
| C6—C7—H7 | 126.5000 | N50—C51—C52 | 108.2 (11) |
| C7—C8—C9 | 106.7 (11) | C54—C51—C52 | 112.7 (12) |
| C7—C8—H8 | 126.7000 | N50—C51—H51 | 108.1000 |
| C9—C8—H8 | 126.7000 | C54—C51—H51 | 108.1000 |
| N2—C9—C10 | 124.6 (13) | C52—C51—H51 | 108.1000 |
| N2—C9—C8 | 111.5 (11) | O51—C52—O52 | 126.7 (17) |
| C10—C9—C8 | 123.9 (13) | O51—C52—C51 | 121.6 (15) |
| C11—C10—C9 | 122.7 (12) | O52—C52—C51 | 111.7 (14) |
| C11—C10—C21 | 118.6 (12) | O52—C53—H53A | 109.5000 |
| C9—C10—C21 | 118.6 (12) | O52—C53—H53B | 109.5000 |
| N3—C11—C10 | 125.9 (12) | H53A—C53—H53B | 109.5000 |
| N3—C11—C12 | 106.9 (11) | O52—C53—H53C | 109.5000 |
| C10—C11—C12 | 127.2 (14) | H53A—C53—H53C | 109.5000 |
| C13—C12—C11 | 107.9 (11) | H53B—C53—H53C | 109.5000 |
| C13—C12—H12 | 126.0000 | C51—C54—H54A | 109.5000 |
| C11—C12—H12 | 126.0000 | C51—C54—H54B | 109.5000 |
| C12—C13—C14 | 108.8 (11) | H54A—C54—H54B | 109.5000 |
| C12—C13—H13 | 125.6000 | C51—C54—H54C | 109.5000 |
| C14—C13—H13 | 125.6000 | H54A—C54—H54C | 109.5000 |
| N3—C14—C15 | 126.1 (13) | H54B—C54—H54C | 109.5000 |
| N3—C14—C13 | 106.8 (11) | O60—C60—N60 | 122.2 (12) |
| C15—C14—C13 | 127.1 (13) | O60—C60—C15 | 122.8 (13) |
| C14—C15—C16 | 129.4 (12) | N60—C60—C15 | 115.0 (11) |
| C14—C15—C60 | 114.9 (12) | N60—C61—C62 | 108.8 (11) |
| C16—C15—C60 | 115.3 (12) | N60—C61—C64 | 108.9 (11) |
| N4—C16—C15 | 123.9 (12) | C62—C61—C64 | 112.2 (12) |
| N4—C16—C17 | 110.5 (12) | N60—C61—H61 | 108.9000 |
| C15—C16—C17 | 125.6 (14) | C62—C61—H61 | 108.9000 |
| C18—C17—C16 | 107.4 (12) | C64—C61—H61 | 108.9000 |
| C18—C17—H17 | 126.3000 | O61—C62—O62 | 121.7 (15) |
| C16—C17—H17 | 126.3000 | O61—C62—C61 | 125.3 (17) |
| C17—C18—C19 | 107.2 (12) | O62—C62—C61 | 112.1 (14) |
| C17—C18—H18 | 126.4000 | O62—C63—H63A | 109.5000 |
| C19—C18—H18 | 126.4000 | O62—C63—H63B | 109.5000 |
| N4—C19—C20 | 125.3 (13) | H63A—C63—H63B | 109.5000 |
| N4—C19—C18 | 110.3 (11) | O62—C63—H63C | 109.5000 |
| C20—C19—C18 | 124.4 (15) | H63A—C63—H63C | 109.5000 |
| C1—C20—C19 | 125.0 (12) | H63B—C63—H63C | 109.5000 |
| C1—C20—C31 | 115.0 (13) | C61—C64—H64A | 109.5000 |
| C19—C20—C31 | 119.8 (13) | C61—C64—H64B | 109.5000 |
| C26—C21—C22 | 118.0 (12) | H64A—C64—H64B | 109.5000 |
| C26—C21—C10 | 122.5 (13) | C61—C64—H64C | 109.5000 |
| C22—C21—C10 | 119.5 (14) | H64A—C64—H64C | 109.5000 |
| C23—C22—C21 | 122.0 (13) | H64B—C64—H64C | 109.5000 |
| C23—C22—H22 | 119.0000 | C1—N1—C4 | 109.8 (10) |
| C21—C22—H22 | 119.0000 | C1—N1—H1 | 125 (8) |
| C24—C23—C22 | 118.4 (14) | C4—N1—H1 | 125 (8) |
| C24—C23—H23 | 120.8000 | C6—N2—C9 | 104.0 (10) |
| C22—C23—H23 | 120.8000 | C11—N3—C14 | 109.6 (10) |
| C25—C24—C23 | 121.1 (15) | C11—N3—H3 | 130 (7) |
| C25—C24—H24 | 119.5000 | C14—N3—H3 | 121 (7) |
| C23—C24—H24 | 119.5000 | C16—N4—C19 | 104.5 (10) |
| C24—C25—C26 | 121.8 (15) | C50—N50—C51 | 123.5 (11) |
| C24—C25—H25 | 119.1000 | C50—N50—H50 | 118.2000 |
| C26—C25—H25 | 119.1000 | C51—N50—H50 | 118.2000 |
| C21—C26—C25 | 118.6 (12) | C60—N60—C61 | 123.6 (11) |
| C21—C26—H26 | 120.7000 | C60—N60—H60 | 118.2000 |
| C25—C26—H26 | 120.7000 | C61—N60—H60 | 118.2000 |
| C32—C31—C36 | 115.6 (14) | C52—O52—C53 | 117.1 (13) |
| C32—C31—C20 | 121.6 (14) | C62—O62—C63 | 117.1 (14) |
| Torsion angles (º) | | | |
| N1—C1—C2—C3 | -0.7 (13) | C23—C24—C25—C26 | 2 (3) |
| C20—C1—C2—C3 | 179.0 (11) | C22—C21—C26—C25 | -3 (2) |
| C1—C2—C3—C4 | -0.7 (13) | C10—C21—C26—C25 | 179.1 (12) |
| C2—C3—C4—C5 | -174.5 (11) | C24—C25—C26—C21 | 2 (2) |
| C2—C3—C4—N1 | 1.8 (13) | C1—C20—C31—C32 | -100.6 (17) |
| N1—C4—C5—C6 | 1.2 (18) | C19—C20—C31—C32 | 75.2 (19) |
| C3—C4—C5—C6 | 176.7 (11) | C1—C20—C31—C36 | 77.7 (18) |
| N1—C4—C5—C50 | -178.6 (11) | C19—C20—C31—C36 | -106.5 (17) |
| C3—C4—C5—C50 | -3.1 (16) | C36—C31—C32—C33 | 1 (3) |
| C4—C5—C6—N2 | 5.1 (19) | C20—C31—C32—C33 | 179.7 (19) |
| C50—C5—C6—N2 | -175.1 (11) | C31—C32—C33—C34 | -4 (4) |
| C4—C5—C6—C7 | -174.6 (11) | C32—C33—C34—C35 | 4 (4) |
| C50—C5—C6—C7 | 5.1 (16) | C33—C34—C35—C36 | -2 (4) |
| N2—C6—C7—C8 | 1.5 (13) | C32—C31—C36—C35 | 1 (3) |
| C5—C6—C7—C8 | -178.7 (11) | C20—C31—C36—C35 | -177.0 (19) |
| C6—C7—C8—C9 | 0.8 (13) | C34—C35—C36—C31 | -1 (4) |
| C7—C8—C9—N2 | -2.8 (14) | C4—C5—C50—O50 | 97.6 (13) |
| C7—C8—C9—C10 | 178.9 (11) | C6—C5—C50—O50 | -82.2 (15) |
| N2—C9—C10—C11 | -4.6 (17) | C4—C5—C50—N50 | -79.0 (13) |
| C8—C9—C10—C11 | 173.5 (10) | C6—C5—C50—N50 | 101.2 (12) |
| N2—C9—C10—C21 | 172.0 (11) | N50—C51—C52—O51 | -146.2 (14) |
| C8—C9—C10—C21 | -10.0 (16) | C54—C51—C52—O51 | -23 (2) |
| C9—C10—C11—N3 | -3.9 (17) | N50—C51—C52—O52 | 35.8 (15) |
| C21—C10—C11—N3 | 179.6 (11) | C54—C51—C52—O52 | 159.4 (12) |
| C9—C10—C11—C12 | -179.9 (10) | C14—C15—C60—O60 | -118.9 (13) |
| C21—C10—C11—C12 | 3.5 (18) | C16—C15—C60—O60 | 67.7 (15) |
| N3—C11—C12—C13 | -1.4 (13) | C14—C15—C60—N60 | 62.4 (14) |
| C10—C11—C12—C13 | 175.2 (11) | C16—C15—C60—N60 | -111.1 (13) |
| C11—C12—C13—C14 | 1.4 (13) | N60—C61—C62—O61 | -36 (2) |
| C12—C13—C14—N3 | -0.8 (12) | C64—C61—C62—O61 | 84 (2) |
| C12—C13—C14—C15 | 178.8 (11) | N60—C61—C62—O62 | 154.3 (13) |
| N3—C14—C15—C16 | 5.1 (19) | C64—C61—C62—O62 | -85.0 (16) |
| C13—C14—C15—C16 | -174.4 (12) | C20—C1—N1—C4 | -177.9 (10) |
| N3—C14—C15—C60 | -167.3 (11) | C2—C1—N1—C4 | 1.9 (12) |
| C13—C14—C15—C60 | 13.2 (17) | C5—C4—N1—C1 | 173.9 (10) |
| C14—C15—C16—N4 | -0.9 (19) | C3—C4—N1—C1 | -2.3 (12) |
| C60—C15—C16—N4 | 171.4 (10) | C5—C6—N2—C9 | 177.1 (10) |
| C14—C15—C16—C17 | 179.1 (11) | C7—C6—N2—C9 | -3.1 (12) |
| C60—C15—C16—C17 | -8.5 (17) | C10—C9—N2—C6 | -178.1 (10) |
| N4—C16—C17—C18 | -0.7 (14) | C8—C9—N2—C6 | 3.6 (12) |
| C15—C16—C17—C18 | 179.2 (12) | C10—C11—N3—C14 | -175.8 (10) |
| C16—C17—C18—C19 | -0.9 (14) | C12—C11—N3—C14 | 0.9 (12) |
| C17—C18—C19—N4 | 2.2 (14) | C15—C14—N3—C11 | -179.7 (11) |
| C17—C18—C19—C20 | -178.1 (11) | C13—C14—N3—C11 | -0.1 (12) |
| N1—C1—C20—C19 | -2.1 (18) | C15—C16—N4—C19 | -177.9 (10) |
| C2—C1—C20—C19 | 178.3 (12) | C17—C16—N4—C19 | 2.0 (12) |
| N1—C1—C20—C31 | 173.4 (11) | C20—C19—N4—C16 | 177.8 (10) |
| C2—C1—C20—C31 | -6.2 (18) | C18—C19—N4—C16 | -2.6 (12) |
| N4—C19—C20—C1 | 1.0 (18) | O50—C50—N50—C51 | -7.1 (18) |
| C18—C19—C20—C1 | -178.6 (11) | C5—C50—N50—C51 | 169.4 (11) |
| N4—C19—C20—C31 | -174.3 (11) | C54—C51—N50—C50 | 95.2 (14) |
| C18—C19—C20—C31 | 6.1 (17) | C52—C51—N50—C50 | -140.3 (12) |
| C11—C10—C21—C26 | -57.6 (17) | O60—C60—N60—C61 | -0.5 (19) |
| C9—C10—C21—C26 | 125.7 (13) | C15—C60—N60—C61 | 178.2 (10) |
| C11—C10—C21—C22 | 125.0 (12) | C62—C61—N60—C60 | -91.0 (14) |
| C9—C10—C21—C22 | -51.7 (16) | C64—C61—N60—C60 | 146.3 (12) |
| C26—C21—C22—C23 | 2 (2) | O51—C52—O52—C53 | 4 (2) |
| C10—C21—C22—C23 | 179.3 (13) | C51—C52—O52—C53 | -177.9 (11) |
| C21—C22—C23—C24 | 2 (3) | O61—C62—O62—C63 | 7 (3) |
| C22—C23—C24—C25 | -4 (3) | C61—C62—O62—C63 | 176.5 (14) |

Table S8. Geometric parameters (Å, º) for **2**

| Distances (*Å)* | | | |
| --- | --- | --- | --- |
| C1—N1 | 1.31 (4) | C101—N101 | 1.41 (5) |
| C1—C20 | 1.40 (5) | C101—C102 | 1.48 (5) |
| C1—C2 | 1.43 (5) | C102—C103 | 1.35 (5) |
| C2—C3 | 1.32 (5) | C103—C104 | 1.37 (5) |
| C1S—Cl13 | 1.69 (3) | C104—N101 | 1.34 (4) |
| C1S—Cl12 | 1.69 (3) | C104—C105 | 1.34 (5) |
| C1S—Cl11 | 1.71 (3) | C105—C106 | 1.42 (5) |
| C3—C4 | 1.44 (5) | C105—Br15 | 1.90 (4) |
| C2S—Cl22 | 1.66 (5) | C106—C107 | 1.35 (5) |
| C2S—Cl21 | 1.72 (5) | C106—N102 | 1.38 (4) |
| C2S—Cl23 | 1.86 (5) | C107—C108 | 1.32 (5) |
| C4—N1 | 1.36 (4) | C108—C109 | 1.46 (5) |
| C4—C5 | 1.45 (5) | C109—C110 | 1.32 (5) |
| C5—C6 | 1.34 (5) | C109—N102 | 1.36 (4) |
| C5—Br5 | 1.88 (4) | C110—C111 | 1.44 (5) |
| C6—N2 | 1.41 (4) | C110—C131 | 1.53 (6) |
| C6—C7 | 1.45 (5) | C111—N103 | 1.37 (4) |
| C7—C8 | 1.38 (5) | C111—C112 | 1.38 (4) |
| C8—C9 | 1.36 (5) | C112—C113 | 1.36 (5) |
| C9—N2 | 1.39 (4) | C113—C114 | 1.47 (5) |
| C9—C10 | 1.41 (5) | C114—C115 | 1.33 (5) |
| C10—C11 | 1.32 (5) | C114—N103 | 1.36 (4) |
| C10—C31 | 1.46 (5) | C115—C116 | 1.45 (4) |
| C11—N3 | 1.36 (4) | C115—Pd2 | 1.99 (3) |
| C11—C12 | 1.51 (4) | C116—N104 | 1.35 (4) |
| C12—C13 | 1.28 (5) | C116—C117 | 1.42 (5) |
| C13—C14 | 1.36 (5) | C117—C118 | 1.35 (4) |
| C14—N3 | 1.41 (4) | C118—C119 | 1.42 (5) |
| C14—C15 | 1.45 (5) | C119—N104 | 1.36 (4) |
| C15—C16 | 1.37 (4) | C119—C120 | 1.40 (4) |
| C15—Pd1 | 1.96 (3) | C120—C121 | 1.49 (5) |
| C16—N4 | 1.37 (4) | C121—C122 | 1.27 (6) |
| C16—C17 | 1.44 (5) | C121—C126 | 1.30 (6) |
| C17—C18 | 1.35 (4) | C122—C123 | 1.27 (6) |
| C18—C19 | 1.41 (5) | C123—C124 | 1.43 (7) |
| C19—N4 | 1.33 (4) | C124—C125 | 1.36 (6) |
| C19—C20 | 1.45 (4) | C125—C126 | 1.33 (6) |
| C20—C21 | 1.51 (5) | C131—C132 | 1.38 (6) |
| C21—C26 | 1.36 (5) | C131—C136 | 1.39 (7) |
| C21—C22 | 1.48 (6) | C132—C133 | 1.17 (6) |
| C22—C23 | 1.43 (6) | C133—C134 | 1.47 (7) |
| C23—C24 | 1.19 (6) | C134—C135 | 1.22 (7) |
| C24—C25 | 1.33 (6) | C135—C136 | 1.51 (7) |
| C25—C26 | 1.41 (6) | C141—C142 | 1.36 (5) |
| C31—C36 | 1.32 (6) | C141—C146 | 1.37 (4) |
| C31—C32 | 1.33 (6) | C141—P181 | 1.80 (4) |
| C32—C33 | 1.52 (6) | C142—C143 | 1.37 (4) |
| C33—C34 | 1.25 (6) | C143—C144 | 1.43 (5) |
| C34—C35 | 1.45 (6) | C144—C145 | 1.38 (5) |
| C35—C36 | 1.32 (6) | C145—C146 | 1.36 (4) |
| C41—C42 | 1.38 (4) | C151—C156 | 1.39 (5) |
| C41—C46 | 1.45 (5) | C151—C152 | 1.40 (5) |
| C41—P81 | 1.84 (4) | C151—P181 | 1.88 (3) |
| C42—C43 | 1.42 (5) | C152—C153 | 1.39 (5) |
| C43—C44 | 1.41 (6) | C153—C154 | 1.43 (6) |
| C44—C45 | 1.28 (6) | C154—C155 | 1.30 (6) |
| C45—C46 | 1.34 (6) | C155—C156 | 1.27 (5) |
| C51—C52 | 1.39 (4) | C161—C162 | 1.29 (5) |
| C51—C56 | 1.43 (5) | C161—C166 | 1.36 (5) |
| C51—P81 | 1.79 (3) | C161—P182 | 1.83 (4) |
| C52—C53 | 1.37 (4) | C162—C163 | 1.43 (6) |
| C53—C54 | 1.26 (5) | C163—C164 | 1.36 (6) |
| C54—C55 | 1.40 (5) | C164—C165 | 1.24 (6) |
| C55—C56 | 1.36 (4) | C165—C166 | 1.40 (6) |
| C61—C62 | 1.35 (5) | C171—C176 | 1.31 (5) |
| C61—C66 | 1.42 (5) | C171—C172 | 1.42 (5) |
| C61—P82 | 1.78 (3) | C171—P182 | 1.78 (4) |
| C62—C63 | 1.35 (4) | C172—C173 | 1.41 (5) |
| C63—C64 | 1.35 (5) | C173—C174 | 1.27 (5) |
| C64—C65 | 1.39 (6) | C174—C175 | 1.35 (6) |
| C65—C66 | 1.32 (5) | C175—C176 | 1.46 (6) |
| C71—C76 | 1.28 (5) | C181—C191 | 1.35 (5) |
| C71—C72 | 1.44 (5) | C181—C182 | 1.36 (5) |
| C71—P82 | 1.78 (4) | C182—C183 | 1.32 (5) |
| C72—C73 | 1.43 (6) | C183—C184 | 1.43 (4) |
| C73—C74 | 1.15 (7) | C184—C192 | 1.40 (4) |
| C74—C75 | 1.47 (7) | C184—P181 | 1.84 (3) |
| C75—C76 | 1.43 (6) | C185—C186 | 1.37 (5) |
| C81—C82 | 1.34 (4) | C185—C193 | 1.42 (5) |
| C81—C93 | 1.37 (5) | C185—P182 | 1.80 (3) |
| C82—C83 | 1.45 (5) | C186—C187 | 1.42 (6) |
| C83—C84 | 1.34 (5) | C187—C188 | 1.31 (5) |
| C84—C92 | 1.38 (4) | C188—C194 | 1.37 (5) |
| C84—P81 | 1.84 (4) | C189—C194 | 1.52 (5) |
| C85—C86 | 1.39 (5) | C189—C196 | 1.52 (5) |
| C85—C91 | 1.39 (5) | C189—C195 | 1.53 (5) |
| C85—P82 | 1.77 (4) | C189—C191 | 1.53 (5) |
| C86—C87 | 1.40 (5) | C191—C192 | 1.40 (5) |
| C87—C88 | 1.41 (5) | C192—O190 | 1.37 (4) |
| C88—C90 | 1.37 (5) | C193—O190 | 1.33 (4) |
| C89—C90 | 1.50 (5) | C193—C194 | 1.39 (5) |
| C89—C94 | 1.52 (6) | Br1—Pd1 | 2.460 (5) |
| C89—C93 | 1.53 (5) | Br10—Pd2 | 2.447 (5) |
| C89—C95 | 1.53 (5) | O80—P82 | 1.46 (2) |
| C90—C91 | 1.39 (5) | O80—Pd1 | 2.151 (19) |
| C91—O90 | 1.38 (4) | O180—P182 | 1.50 (2) |
| C92—O90 | 1.39 (4) | O180—Pd2 | 2.20 (2) |
| C92—C93 | 1.40 (5) | P81—Pd1 | 2.258 (10) |
| C101—C120 | 1.30 (5) | P181—Pd2 | 2.294 (10) |
| Angles (º) | | | |
| N1—C1—C20 | 127 (3) | C111—C110—C131 | 114 (3) |
| N1—C1—C2 | 113 (4) | N103—C111—C112 | 111 (3) |
| C20—C1—C2 | 120 (4) | N103—C111—C110 | 122 (3) |
| C3—C2—C1 | 103 (4) | C112—C111—C110 | 126 (4) |
| Cl13—C1S—Cl12 | 112 (3) | C113—C112—C111 | 107 (3) |
| Cl13—C1S—Cl11 | 95 (3) | C112—C113—C114 | 108 (3) |
| Cl12—C1S—Cl11 | 99 (3) | C115—C114—N103 | 129 (3) |
| C2—C3—C4 | 111 (4) | C115—C114—C113 | 124 (3) |
| Cl22—C2S—Cl21 | 118 (3) | N103—C114—C113 | 107 (3) |
| Cl22—C2S—Cl23 | 105 (3) | C114—C115—C116 | 125 (3) |
| Cl21—C2S—Cl23 | 104 (3) | C114—C115—Pd2 | 119 (3) |
| N1—C4—C3 | 106 (4) | C116—C115—Pd2 | 116 (3) |
| N1—C4—C5 | 123 (3) | N104—C116—C117 | 110 (3) |
| C3—C4—C5 | 131 (4) | N104—C116—C115 | 126 (3) |
| C6—C5—C4 | 128 (4) | C117—C116—C115 | 124 (3) |
| C6—C5—Br5 | 117 (3) | C118—C117—C116 | 105 (3) |
| C4—C5—Br5 | 114 (3) | C117—C118—C119 | 109 (3) |
| C5—C6—N2 | 126 (4) | N104—C119—C120 | 123 (3) |
| C5—C6—C7 | 125 (3) | N104—C119—C118 | 107 (3) |
| N2—C6—C7 | 109 (3) | C120—C119—C118 | 130 (4) |
| C8—C7—C6 | 107 (3) | C101—C120—C119 | 129 (4) |
| C9—C8—C7 | 108 (4) | C101—C120—C121 | 117 (3) |
| C8—C9—N2 | 113 (3) | C119—C120—C121 | 114 (4) |
| C8—C9—C10 | 126 (4) | C122—C121—C126 | 109 (5) |
| N2—C9—C10 | 122 (4) | C122—C121—C120 | 126 (5) |
| C11—C10—C9 | 127 (4) | C126—C121—C120 | 125 (4) |
| C11—C10—C31 | 117 (3) | C123—C122—C121 | 129 (6) |
| C9—C10—C31 | 116 (4) | C122—C123—C124 | 123 (5) |
| C10—C11—N3 | 128 (3) | C125—C124—C123 | 107 (5) |
| C10—C11—C12 | 129 (4) | C126—C125—C124 | 122 (6) |
| N3—C11—C12 | 103 (3) | C121—C126—C125 | 129 (6) |
| C13—C12—C11 | 109 (3) | C132—C131—C136 | 116 (5) |
| C12—C13—C14 | 112 (4) | C132—C131—C110 | 122 (5) |
| C13—C14—N3 | 107 (3) | C136—C131—C110 | 122 (4) |
| C13—C14—C15 | 130 (3) | C133—C132—C131 | 126 (7) |
| N3—C14—C15 | 123 (3) | C132—C133—C134 | 121 (6) |
| C16—C15—C14 | 127 (3) | C135—C134—C133 | 118 (6) |
| C16—C15—Pd1 | 121 (3) | C134—C135—C136 | 122 (6) |
| C14—C15—Pd1 | 112 (2) | C131—C136—C135 | 116 (5) |
| C15—C16—N4 | 125 (3) | C142—C141—C146 | 116 (3) |
| C15—C16—C17 | 123 (3) | C142—C141—P181 | 124 (3) |
| N4—C16—C17 | 111 (3) | C146—C141—P181 | 119 (3) |
| C18—C17—C16 | 105 (3) | C141—C142—C143 | 123 (3) |
| C17—C18—C19 | 106 (4) | C142—C143—C144 | 119 (4) |
| N4—C19—C18 | 113 (3) | C145—C144—C143 | 119 (3) |
| N4—C19—C20 | 126 (4) | C146—C145—C144 | 119 (3) |
| C18—C19—C20 | 121 (4) | C145—C146—C141 | 124 (4) |
| C1—C20—C19 | 123 (4) | C156—C151—C152 | 120 (3) |
| C1—C20—C21 | 121 (3) | C156—C151—P181 | 120 (3) |
| C19—C20—C21 | 116 (3) | C152—C151—P181 | 120 (3) |
| C26—C21—C22 | 117 (4) | C153—C152—C151 | 118 (4) |
| C26—C21—C20 | 121 (4) | C152—C153—C154 | 116 (5) |
| C22—C21—C20 | 121 (4) | C155—C154—C153 | 121 (5) |
| C23—C22—C21 | 111 (4) | C156—C155—C154 | 124 (5) |
| C24—C23—C22 | 128 (5) | C155—C156—C151 | 119 (4) |
| C23—C24—C25 | 123 (5) | C162—C161—C166 | 123 (4) |
| C24—C25—C26 | 117 (5) | C162—C161—P182 | 119 (4) |
| C21—C26—C25 | 123 (5) | C166—C161—P182 | 117 (3) |
| C36—C31—C32 | 113 (5) | C161—C162—C163 | 116 (5) |
| C36—C31—C10 | 125 (5) | C164—C163—C162 | 118 (5) |
| C32—C31—C10 | 122 (5) | C165—C164—C163 | 122 (5) |
| C31—C32—C33 | 119 (5) | C164—C165—C166 | 121 (5) |
| C34—C33—C32 | 115 (5) | C161—C166—C165 | 117 (5) |
| C33—C34—C35 | 129 (5) | C176—C171—C172 | 121 (4) |
| C36—C35—C34 | 104 (5) | C176—C171—P182 | 124 (3) |
| C31—C36—C35 | 137 (6) | C172—C171—P182 | 115 (3) |
| C42—C41—C46 | 119 (4) | C173—C172—C171 | 116 (4) |
| C42—C41—P81 | 121 (3) | C174—C173—C172 | 125 (4) |
| C46—C41—P81 | 120 (3) | C173—C174—C175 | 119 (5) |
| C41—C42—C43 | 119 (4) | C174—C175—C176 | 120 (4) |
| C44—C43—C42 | 117 (4) | C171—C176—C175 | 119 (4) |
| C45—C44—C43 | 124 (5) | C191—C181—C182 | 122 (4) |
| C44—C45—C46 | 121 (6) | C183—C182—C181 | 120 (4) |
| C45—C46—C41 | 119 (4) | C182—C183—C184 | 122 (4) |
| C52—C51—C56 | 116 (3) | C192—C184—C183 | 116 (3) |
| C52—C51—P81 | 122 (3) | C192—C184—P181 | 122 (3) |
| C56—C51—P81 | 121 (3) | C183—C184—P181 | 122 (3) |
| C53—C52—C51 | 124 (4) | C186—C185—C193 | 118 (3) |
| C54—C53—C52 | 116 (4) | C186—C185—P182 | 127 (3) |
| C53—C54—C55 | 127 (4) | C193—C185—P182 | 114 (3) |
| C56—C55—C54 | 117 (4) | C185—C186—C187 | 120 (4) |
| C55—C56—C51 | 119 (3) | C188—C187—C186 | 118 (5) |
| C62—C61—C66 | 113 (3) | C187—C188—C194 | 126 (4) |
| C62—C61—P82 | 124 (3) | C194—C189—C196 | 111 (4) |
| C66—C61—P82 | 122 (3) | C194—C189—C195 | 106 (3) |
| C63—C62—C61 | 127 (4) | C196—C189—C195 | 110 (4) |
| C62—C63—C64 | 117 (4) | C194—C189—C191 | 113 (3) |
| C63—C64—C65 | 121 (4) | C196—C189—C191 | 109 (3) |
| C66—C65—C64 | 119 (5) | C195—C189—C191 | 108 (4) |
| C65—C66—C61 | 123 (5) | C181—C191—C192 | 119 (4) |
| C76—C71—C72 | 116 (4) | C181—C191—C189 | 126 (3) |
| C76—C71—P82 | 124 (3) | C192—C191—C189 | 115 (3) |
| C72—C71—P82 | 119 (3) | O190—C192—C184 | 112 (3) |
| C73—C72—C71 | 115 (5) | O190—C192—C191 | 127 (3) |
| C74—C73—C72 | 129 (6) | C184—C192—C191 | 121 (3) |
| C73—C74—C75 | 119 (6) | O190—C193—C194 | 123 (3) |
| C76—C75—C74 | 113 (5) | O190—C193—C185 | 116 (3) |
| C71—C76—C75 | 127 (4) | C194—C193—C185 | 121 (4) |
| C82—C81—C93 | 123 (4) | C188—C194—C193 | 116 (4) |
| C81—C82—C83 | 120 (4) | C188—C194—C189 | 123 (3) |
| C84—C83—C82 | 118 (4) | C193—C194—C189 | 121 (4) |
| C83—C84—C92 | 120 (4) | C1—N1—C4 | 108 (3) |
| C83—C84—P81 | 124 (3) | C9—N2—C6 | 104 (3) |
| C92—C84—P81 | 116 (3) | C11—N3—C14 | 110 (3) |
| C86—C85—C91 | 117 (4) | C19—N4—C16 | 104 (3) |
| C86—C85—P82 | 122 (3) | C104—N101—C101 | 113 (3) |
| C91—C85—P82 | 120 (3) | C109—N102—C106 | 107 (3) |
| C85—C86—C87 | 117 (4) | C114—N103—C111 | 107 (3) |
| C86—C87—C88 | 120 (4) | C116—N104—C119 | 108 (3) |
| C90—C88—C87 | 125 (4) | P82—O80—Pd1 | 136.2 (13) |
| C90—C89—C94 | 110 (4) | C91—O90—C92 | 122 (3) |
| C90—C89—C93 | 107 (3) | P182—O180—Pd2 | 131.1 (12) |
| C94—C89—C93 | 111 (4) | C193—O190—C192 | 118 (3) |
| C90—C89—C95 | 107 (4) | C51—P81—C84 | 106.7 (15) |
| C94—C89—C95 | 111 (4) | C51—P81—C41 | 104.4 (17) |
| C93—C89—C95 | 110 (3) | C84—P81—C41 | 99.8 (18) |
| C88—C90—C91 | 110 (4) | C51—P81—Pd1 | 111.5 (11) |
| C88—C90—C89 | 124 (4) | C84—P81—Pd1 | 115.4 (13) |
| C91—C90—C89 | 124 (4) | C41—P81—Pd1 | 117.7 (13) |
| O90—C91—C85 | 111 (3) | O80—P82—C85 | 114.0 (15) |
| O90—C91—C90 | 119 (3) | O80—P82—C61 | 110.0 (16) |
| C85—C91—C90 | 130 (4) | C85—P82—C61 | 103.3 (18) |
| C84—C92—O90 | 119 (3) | O80—P82—C71 | 110.2 (16) |
| C84—C92—C93 | 123 (4) | C85—P82—C71 | 109 (2) |
| O90—C92—C93 | 118 (3) | C61—P82—C71 | 109.7 (17) |
| C81—C93—C92 | 116 (4) | C141—P181—C184 | 108.2 (15) |
| C81—C93—C89 | 119 (4) | C141—P181—C151 | 105.0 (17) |
| C92—C93—C89 | 125 (3) | C184—P181—C151 | 102.2 (16) |
| C120—C101—N101 | 129 (3) | C141—P181—Pd2 | 113.1 (12) |
| C120—C101—C102 | 131 (4) | C184—P181—Pd2 | 112.1 (12) |
| N101—C101—C102 | 100 (3) | C151—P181—Pd2 | 115.5 (11) |
| C103—C102—C101 | 109 (4) | O180—P182—C171 | 113.5 (17) |
| C102—C103—C104 | 109 (4) | O180—P182—C185 | 116.7 (14) |
| N101—C104—C105 | 126 (4) | C171—P182—C185 | 102.0 (17) |
| N101—C104—C103 | 108 (4) | O180—P182—C161 | 113.2 (15) |
| C105—C104—C103 | 125 (4) | C171—P182—C161 | 104.2 (18) |
| C104—C105—C106 | 128 (4) | C185—P182—C161 | 105.8 (17) |
| C104—C105—Br15 | 117 (3) | C15—Pd1—O80 | 175.7 (11) |
| C106—C105—Br15 | 114 (3) | C15—Pd1—P81 | 89.1 (10) |
| C107—C106—N102 | 109 (4) | O80—Pd1—P81 | 94.6 (6) |
| C107—C106—C105 | 129 (4) | C15—Pd1—Br1 | 86.9 (10) |
| N102—C106—C105 | 122 (4) | O80—Pd1—Br1 | 89.5 (6) |
| C108—C107—C106 | 112 (4) | P81—Pd1—Br1 | 175.6 (3) |
| C107—C108—C109 | 105 (4) | C115—Pd2—O180 | 172.9 (12) |
| C110—C109—N102 | 129 (4) | C115—Pd2—P181 | 88.4 (11) |
| C110—C109—C108 | 123 (4) | O180—Pd2—P181 | 94.2 (6) |
| N102—C109—C108 | 108 (3) | C115—Pd2—Br10 | 87.4 (10) |
| C109—C110—C111 | 125 (4) | O180—Pd2—Br10 | 90.4 (6) |
| C109—C110—C131 | 120 (4) | P181—Pd2—Br10 | 174.6 (3) |
| Torsion angles (º) | | | |
| N1—C1—C2—C3 | 2 (5) | C122—C123—C124—C125 | -9 (8) |
| C20—C1—C2—C3 | 178 (4) | C123—C124—C125—C126 | 10 (8) |
| C1—C2—C3—C4 | 0 (5) | C122—C121—C126—C125 | 4 (9) |
| C2—C3—C4—N1 | -2 (5) | C120—C121—C126—C125 | -178 (5) |
| C2—C3—C4—C5 | -179 (5) | C124—C125—C126—C121 | -9 (11) |
| N1—C4—C5—C6 | 8 (7) | C109—C110—C131—C132 | -86 (6) |
| C3—C4—C5—C6 | -177 (4) | C111—C110—C131—C132 | 101 (5) |
| N1—C4—C5—Br5 | -177 (3) | C109—C110—C131—C136 | 103 (6) |
| C3—C4—C5—Br5 | -1 (6) | C111—C110—C131—C136 | -71 (6) |
| C4—C5—C6—N2 | -1 (7) | C136—C131—C132—C133 | -11 (11) |
| Br5—C5—C6—N2 | -177 (3) | C110—C131—C132—C133 | 177 (7) |
| C4—C5—C6—C7 | 171 (4) | C131—C132—C133—C134 | 13 (13) |
| Br5—C5—C6—C7 | -5 (6) | C132—C133—C134—C135 | -11 (12) |
| C5—C6—C7—C8 | -176 (4) | C133—C134—C135—C136 | 6 (11) |
| N2—C6—C7—C8 | -3 (4) | C132—C131—C136—C135 | 5 (9) |
| C6—C7—C8—C9 | 3 (4) | C110—C131—C136—C135 | 177 (5) |
| C7—C8—C9—N2 | -1 (5) | C134—C135—C136—C131 | -4 (11) |
| C7—C8—C9—C10 | 178 (4) | C146—C141—C142—C143 | 0 (6) |
| C8—C9—C10—C11 | 178 (4) | P181—C141—C142—C143 | -171 (3) |
| N2—C9—C10—C11 | -3 (7) | C141—C142—C143—C144 | -3 (6) |
| C8—C9—C10—C31 | -3 (6) | C142—C143—C144—C145 | 5 (7) |
| N2—C9—C10—C31 | 176 (4) | C143—C144—C145—C146 | -3 (7) |
| C9—C10—C11—N3 | 3 (7) | C144—C145—C146—C141 | 0 (7) |
| C31—C10—C11—N3 | -176 (4) | C142—C141—C146—C145 | 2 (6) |
| C9—C10—C11—C12 | -174 (4) | P181—C141—C146—C145 | 174 (3) |
| C31—C10—C11—C12 | 7 (7) | C156—C151—C152—C153 | -5 (6) |
| C10—C11—C12—C13 | 177 (4) | P181—C151—C152—C153 | 171 (4) |
| N3—C11—C12—C13 | -1 (4) | C151—C152—C153—C154 | 2 (7) |
| C11—C12—C13—C14 | 3 (5) | C152—C153—C154—C155 | 5 (8) |
| C12—C13—C14—N3 | -4 (5) | C153—C154—C155—C156 | -9 (8) |
| C12—C13—C14—C15 | 179 (4) | C154—C155—C156—C151 | 7 (8) |
| C13—C14—C15—C16 | 174 (4) | C152—C151—C156—C155 | 0 (6) |
| N3—C14—C15—C16 | -3 (6) | P181—C151—C156—C155 | -175 (3) |
| C13—C14—C15—Pd1 | -7 (5) | C166—C161—C162—C163 | -12 (9) |
| N3—C14—C15—Pd1 | 177 (3) | P182—C161—C162—C163 | 177 (5) |
| C14—C15—C16—N4 | 7 (6) | C161—C162—C163—C164 | 18 (10) |
| Pd1—C15—C16—N4 | -172 (2) | C162—C163—C164—C165 | -14 (11) |
| C14—C15—C16—C17 | 174 (3) | C163—C164—C165—C166 | 3 (13) |
| Pd1—C15—C16—C17 | -5 (5) | C162—C161—C166—C165 | 2 (9) |
| C15—C16—C17—C18 | -170 (3) | P182—C161—C166—C165 | 172 (6) |
| N4—C16—C17—C18 | -2 (4) | C164—C165—C166—C161 | 4 (12) |
| C16—C17—C18—C19 | -1 (4) | C176—C171—C172—C173 | 5 (5) |
| C17—C18—C19—N4 | 3 (5) | P182—C171—C172—C173 | -178 (2) |
| C17—C18—C19—C20 | -180 (3) | C171—C172—C173—C174 | 3 (6) |
| N1—C1—C20—C19 | -3 (6) | C172—C173—C174—C175 | -6 (7) |
| C2—C1—C20—C19 | -179 (4) | C173—C174—C175—C176 | 2 (7) |
| N1—C1—C20—C21 | 175 (4) | C172—C171—C176—C175 | -8 (6) |
| C2—C1—C20—C21 | 0 (6) | P182—C171—C176—C175 | 175 (3) |
| N4—C19—C20—C1 | -2 (6) | C174—C175—C176—C171 | 5 (7) |
| C18—C19—C20—C1 | -179 (4) | C191—C181—C182—C183 | 6 (6) |
| N4—C19—C20—C21 | 180 (4) | C181—C182—C183—C184 | -2 (6) |
| C18—C19—C20—C21 | 3 (5) | C182—C183—C184—C192 | -7 (5) |
| C1—C20—C21—C26 | -89 (5) | C182—C183—C184—P181 | 172 (3) |
| C19—C20—C21—C26 | 89 (5) | C193—C185—C186—C187 | 0 (6) |
| C1—C20—C21—C22 | 82 (5) | P182—C185—C186—C187 | 167 (4) |
| C19—C20—C21—C22 | -100 (4) | C185—C186—C187—C188 | -6 (8) |
| C26—C21—C22—C23 | -11 (7) | C186—C187—C188—C194 | 7 (9) |
| C20—C21—C22—C23 | 178 (4) | C182—C181—C191—C192 | -2 (6) |
| C21—C22—C23—C24 | 10 (9) | C182—C181—C191—C189 | -179 (4) |
| C22—C23—C24—C25 | -7 (10) | C194—C189—C191—C181 | 169 (3) |
| C23—C24—C25—C26 | 4 (10) | C196—C189—C191—C181 | 46 (5) |
| C22—C21—C26—C25 | 10 (8) | C195—C189—C191—C181 | -74 (5) |
| C20—C21—C26—C25 | -179 (5) | C194—C189—C191—C192 | -8 (5) |
| C24—C25—C26—C21 | -6 (9) | C196—C189—C191—C192 | -132 (3) |
| C11—C10—C31—C36 | 72 (6) | C195—C189—C191—C192 | 109 (4) |
| C9—C10—C31—C36 | -107 (5) | C183—C184—C192—O190 | -169 (3) |
| C11—C10—C31—C32 | -104 (5) | P181—C184—C192—O190 | 12 (4) |
| C9—C10—C31—C32 | 77 (6) | C183—C184—C192—C191 | 11 (5) |
| C36—C31—C32—C33 | -5 (7) | P181—C184—C192—C191 | -167 (3) |
| C10—C31—C32—C33 | 171 (4) | C181—C191—C192—O190 | 173 (3) |
| C31—C32—C33—C34 | 9 (8) | C189—C191—C192—O190 | -9 (5) |
| C32—C33—C34—C35 | -17 (8) | C181—C191—C192—C184 | -7 (5) |
| C33—C34—C35—C36 | 20 (7) | C189—C191—C192—C184 | 170 (3) |
| C32—C31—C36—C35 | 13 (9) | C186—C185—C193—O190 | -175 (3) |
| C10—C31—C36—C35 | -163 (5) | P182—C185—C193—O190 | 17 (4) |
| C34—C35—C36—C31 | -18 (8) | C186—C185—C193—C194 | 5 (5) |
| C46—C41—C42—C43 | 2 (5) | P182—C185—C193—C194 | -163 (3) |
| P81—C41—C42—C43 | 174 (3) | C187—C188—C194—C193 | -2 (7) |
| C41—C42—C43—C44 | 0 (6) | C187—C188—C194—C189 | -177 (5) |
| C42—C43—C44—C45 | 7 (8) | O190—C193—C194—C188 | 176 (3) |
| C43—C44—C45—C46 | -15 (10) | C185—C193—C194—C188 | -4 (5) |
| C44—C45—C46—C41 | 16 (9) | O190—C193—C194—C189 | -9 (6) |
| C42—C41—C46—C45 | -9 (7) | C185—C193—C194—C189 | 171 (3) |
| P81—C41—C46—C45 | 179 (4) | C196—C189—C194—C188 | -46 (5) |
| C56—C51—C52—C53 | 3 (5) | C195—C189—C194—C188 | 73 (5) |
| P81—C51—C52—C53 | -172 (3) | C191—C189—C194—C188 | -169 (4) |
| C51—C52—C53—C54 | -3 (6) | C196—C189—C194—C193 | 139 (4) |
| C52—C53—C54—C55 | 3 (6) | C195—C189—C194—C193 | -102 (4) |
| C53—C54—C55—C56 | -4 (6) | C191—C189—C194—C193 | 17 (5) |
| C54—C55—C56—C51 | 3 (5) | C20—C1—N1—C4 | -179 (4) |
| C52—C51—C56—C55 | -3 (5) | C2—C1—N1—C4 | -4 (5) |
| P81—C51—C56—C55 | 172 (3) | C3—C4—N1—C1 | 3 (4) |
| C66—C61—C62—C63 | 2 (6) | C5—C4—N1—C1 | -180 (4) |
| P82—C61—C62—C63 | 171 (3) | C8—C9—N2—C6 | 0 (4) |
| C61—C62—C63—C64 | -1 (6) | C10—C9—N2—C6 | -179 (4) |
| C62—C63—C64—C65 | 7 (7) | C5—C6—N2—C9 | 175 (4) |
| C63—C64—C65—C66 | -12 (8) | C7—C6—N2—C9 | 2 (4) |
| C64—C65—C66—C61 | 13 (8) | C10—C11—N3—C14 | -179 (4) |
| C62—C61—C66—C65 | -8 (7) | C12—C11—N3—C14 | -2 (4) |
| P82—C61—C66—C65 | -178 (4) | C13—C14—N3—C11 | 4 (4) |
| C76—C71—C72—C73 | -6 (8) | C15—C14—N3—C11 | -179 (3) |
| P82—C71—C72—C73 | 178 (5) | C18—C19—N4—C16 | -4 (4) |
| C71—C72—C73—C74 | 10 (13) | C20—C19—N4—C16 | 179 (4) |
| C72—C73—C74—C75 | -6 (14) | C15—C16—N4—C19 | 172 (3) |
| C73—C74—C75—C76 | -1 (11) | C17—C16—N4—C19 | 3 (4) |
| C72—C71—C76—C75 | 0 (9) | C105—C104—N101—C101 | 178 (4) |
| P82—C71—C76—C75 | 176 (5) | C103—C104—N101—C101 | 3 (4) |
| C74—C75—C76—C71 | 4 (9) | C120—C101—N101—C104 | 174 (4) |
| C93—C81—C82—C83 | -4 (6) | C102—C101—N101—C104 | -5 (4) |
| C81—C82—C83—C84 | 8 (5) | C110—C109—N102—C106 | -176 (5) |
| C82—C83—C84—C92 | -4 (5) | C108—C109—N102—C106 | -2 (5) |
| C82—C83—C84—P81 | -173 (2) | C107—C106—N102—C109 | 3 (5) |
| C91—C85—C86—C87 | 0 (5) | C105—C106—N102—C109 | -178 (4) |
| P82—C85—C86—C87 | -169 (3) | C115—C114—N103—C111 | 176 (4) |
| C85—C86—C87—C88 | 6 (6) | C113—C114—N103—C111 | 0 (4) |
| C86—C87—C88—C90 | -8 (7) | C112—C111—N103—C114 | 3 (4) |
| C87—C88—C90—C91 | 4 (7) | C110—C111—N103—C114 | 177 (3) |
| C87—C88—C90—C89 | 167 (4) | C117—C116—N104—C119 | 0 (4) |
| C94—C89—C90—C88 | -63 (5) | C115—C116—N104—C119 | -179 (3) |
| C93—C89—C90—C88 | 176 (4) | C120—C119—N104—C116 | 180 (3) |
| C95—C89—C90—C88 | 58 (5) | C118—C119—N104—C116 | -1 (4) |
| C94—C89—C90—C91 | 98 (5) | C85—C91—O90—C92 | -175 (3) |
| C93—C89—C90—C91 | -23 (5) | C90—C91—O90—C92 | 7 (5) |
| C95—C89—C90—C91 | -141 (4) | C84—C92—O90—C91 | 164 (3) |
| C86—C85—C91—O90 | 179 (3) | C93—C92—O90—C91 | -19 (4) |
| P82—C85—C91—O90 | -12 (4) | C194—C193—O190—C192 | -8 (5) |
| C86—C85—C91—C90 | -4 (6) | C185—C193—O190—C192 | 172 (3) |
| P82—C85—C91—C90 | 165 (3) | C184—C192—O190—C193 | -161 (3) |
| C88—C90—C91—O90 | 179 (4) | C191—C192—O190—C193 | 18 (5) |
| C89—C90—C91—O90 | 16 (6) | C52—C51—P81—C84 | -164 (3) |
| C88—C90—C91—C85 | 2 (6) | C56—C51—P81—C84 | 22 (3) |
| C89—C90—C91—C85 | -161 (4) | C52—C51—P81—C41 | 91 (3) |
| C83—C84—C92—O90 | 173 (3) | C56—C51—P81—C41 | -83 (3) |
| P81—C84—C92—O90 | -17 (4) | C52—C51—P81—Pd1 | -37 (3) |
| C83—C84—C92—C93 | -4 (6) | C56—C51—P81—Pd1 | 148 (3) |
| P81—C84—C92—C93 | 166 (3) | C83—C84—P81—C51 | -94 (3) |
| C82—C81—C93—C92 | -4 (5) | C92—C84—P81—C51 | 97 (3) |
| C82—C81—C93—C89 | 178 (4) | C83—C84—P81—C41 | 14 (3) |
| C84—C92—C93—C81 | 8 (5) | C92—C84—P81—C41 | -155 (3) |
| O90—C92—C93—C81 | -169 (3) | C83—C84—P81—Pd1 | 141 (3) |
| C84—C92—C93—C89 | -175 (4) | C92—C84—P81—Pd1 | -28 (3) |
| O90—C92—C93—C89 | 8 (5) | C42—C41—P81—C51 | 165 (3) |
| C90—C89—C93—C81 | -171 (3) | C46—C41—P81—C51 | -23 (4) |
| C94—C89—C93—C81 | 68 (5) | C42—C41—P81—C84 | 55 (3) |
| C95—C89—C93—C81 | -56 (5) | C46—C41—P81—C84 | -134 (4) |
| C90—C89—C93—C92 | 11 (5) | C42—C41—P81—Pd1 | -71 (3) |
| C94—C89—C93—C92 | -110 (4) | C46—C41—P81—Pd1 | 101 (3) |
| C95—C89—C93—C92 | 127 (4) | Pd1—O80—P82—C85 | 6 (3) |
| C120—C101—C102—C103 | -174 (5) | Pd1—O80—P82—C61 | 121.6 (19) |
| N101—C101—C102—C103 | 6 (4) | Pd1—O80—P82—C71 | -117 (2) |
| C101—C102—C103—C104 | -4 (5) | C86—C85—P82—O80 | -136 (3) |
| C102—C103—C104—N101 | 1 (5) | C91—C85—P82—O80 | 55 (3) |
| C102—C103—C104—C105 | -174 (4) | C86—C85—P82—C61 | 104 (3) |
| N101—C104—C105—C106 | -2 (7) | C91—C85—P82—C61 | -65 (3) |
| C103—C104—C105—C106 | 173 (4) | C86—C85—P82—C71 | -12 (4) |
| N101—C104—C105—Br15 | 179 (3) | C91—C85—P82—C71 | 179 (3) |
| C103—C104—C105—Br15 | -7 (6) | C62—C61—P82—O80 | 35 (3) |
| C104—C105—C106—C107 | -179 (5) | C66—C61—P82—O80 | -157 (3) |
| Br15—C105—C106—C107 | 1 (6) | C62—C61—P82—C85 | 157 (3) |
| C104—C105—C106—N102 | 3 (7) | C66—C61—P82—C85 | -35 (4) |
| Br15—C105—C106—N102 | -178 (3) | C62—C61—P82—C71 | -87 (3) |
| N102—C106—C107—C108 | -4 (5) | C66—C61—P82—C71 | 82 (4) |
| C105—C106—C107—C108 | 177 (4) | C76—C71—P82—O80 | 57 (5) |
| C106—C107—C108—C109 | 3 (5) | C72—C71—P82—O80 | -128 (4) |
| C107—C108—C109—C110 | 174 (4) | C76—C71—P82—C85 | -69 (5) |
| C107—C108—C109—N102 | -1 (5) | C72—C71—P82—C85 | 106 (4) |
| N102—C109—C110—C111 | -5 (7) | C76—C71—P82—C61 | 178 (4) |
| C108—C109—C110—C111 | -178 (4) | C72—C71—P82—C61 | -7 (4) |
| N102—C109—C110—C131 | -177 (4) | C142—C141—P181—C184 | -26 (4) |
| C108—C109—C110—C131 | 9 (7) | C146—C141—P181—C184 | 162 (3) |
| C109—C110—C111—N103 | 3 (6) | C142—C141—P181—C151 | 82 (3) |
| C131—C110—C111—N103 | 176 (4) | C146—C141—P181—C151 | -89 (3) |
| C109—C110—C111—C112 | 177 (4) | C142—C141—P181—Pd2 | -151 (3) |
| C131—C110—C111—C112 | -10 (6) | C146—C141—P181—Pd2 | 38 (3) |
| N103—C111—C112—C113 | -5 (4) | C192—C184—P181—C141 | -93 (3) |
| C110—C111—C112—C113 | -179 (4) | C183—C184—P181—C141 | 89 (3) |
| C111—C112—C113—C114 | 5 (4) | C192—C184—P181—C151 | 157 (3) |
| C112—C113—C114—C115 | -179 (3) | C183—C184—P181—C151 | -22 (3) |
| C112—C113—C114—N103 | -3 (4) | C192—C184—P181—Pd2 | 33 (3) |
| N103—C114—C115—C116 | 1 (6) | C183—C184—P181—Pd2 | -146 (2) |
| C113—C114—C115—C116 | 177 (3) | C156—C151—P181—C141 | -163 (3) |
| N103—C114—C115—Pd2 | -174 (3) | C152—C151—P181—C141 | 22 (3) |
| C113—C114—C115—Pd2 | 1 (5) | C156—C151—P181—C184 | -50 (3) |
| C114—C115—C116—N104 | 1 (6) | C152—C151—P181—C184 | 135 (3) |
| Pd2—C115—C116—N104 | 177 (3) | C156—C151—P181—Pd2 | 72 (3) |
| C114—C115—C116—C117 | -178 (3) | C152—C151—P181—Pd2 | -103 (3) |
| Pd2—C115—C116—C117 | -2 (4) | Pd2—O180—P182—C171 | -122.9 (19) |
| N104—C116—C117—C118 | 0 (4) | Pd2—O180—P182—C185 | -5 (2) |
| C115—C116—C117—C118 | 179 (3) | Pd2—O180—P182—C161 | 119 (2) |
| C116—C117—C118—C119 | -1 (4) | C176—C171—P182—O180 | 154 (3) |
| C117—C118—C119—N104 | 1 (4) | C172—C171—P182—O180 | -23 (3) |
| C117—C118—C119—C120 | -179 (4) | C176—C171—P182—C185 | 28 (4) |
| N101—C101—C120—C119 | 4 (7) | C172—C171—P182—C185 | -149 (3) |
| C102—C101—C120—C119 | -177 (4) | C176—C171—P182—C161 | -82 (4) |
| N101—C101—C120—C121 | -180 (4) | C172—C171—P182—C161 | 101 (3) |
| C102—C101—C120—C121 | 0 (7) | C186—C185—P182—O180 | 134 (3) |
| N104—C119—C120—C101 | -3 (6) | C193—C185—P182—O180 | -59 (3) |
| C118—C119—C120—C101 | 178 (4) | C186—C185—P182—C171 | -102 (4) |
| N104—C119—C120—C121 | -179 (3) | C193—C185—P182—C171 | 65 (3) |
| C118—C119—C120—C121 | 2 (6) | C186—C185—P182—C161 | 7 (4) |
| C101—C120—C121—C122 | -85 (6) | C193—C185—P182—C161 | 174 (3) |
| C119—C120—C121—C122 | 92 (6) | C162—C161—P182—O180 | 120 (4) |
| C101—C120—C121—C126 | 97 (6) | C166—C161—P182—O180 | -52 (4) |
| C119—C120—C121—C126 | -86 (5) | C162—C161—P182—C171 | -4 (4) |
| C126—C121—C122—C123 | -3 (10) | C166—C161—P182—C171 | -175 (4) |
| C120—C121—C122—C123 | 179 (6) | C162—C161—P182—C185 | -111 (4) |
| C121—C122—C123—C124 | 6 (11) | C166—C161—P182—C185 | 78 (4) |

Table S9. Geometric parameters (Å, º) for **3**

| Distances (*Å)* | | | |
| --- | --- | --- | --- |
| C1—N1 | 1.30 (2) | C43—C44 | 1.37 (2) |
| C1—C20 | 1.37 (2) | C44—C45 | 1.36 (2) |
| C1—C2 | 1.48 (2) | C45—C46 | 1.39 (2) |
| C2—C3 | 1.35 (3) | C51—C56 | 1.372 (16) |
| C3—C4 | 1.41 (2) | C51—C52 | 1.399 (16) |
| C4—C5 | 1.31 (2) | C51—P1 | 1.803 (12) |
| C4—N1 | 1.41 (2) | C52—C53 | 1.359 (17) |
| C5—C6 | 1.37 (2) | C53—C54 | 1.373 (18) |
| C5—Br5 | 1.914 (18) | C54—C55 | 1.34 (2) |
| C6—C7 | 1.37 (2) | C55—C56 | 1.373 (19) |
| C6—N2 | 1.42 (2) | C61—C66 | 1.346 (19) |
| C7—C8 | 1.36 (2) | C61—C62 | 1.378 (19) |
| C8—C9 | 1.43 (2) | C61—P2 | 1.819 (14) |
| C9—C10 | 1.34 (2) | C62—C63 | 1.42 (2) |
| C9—N2 | 1.383 (19) | C63—C64 | 1.36 (2) |
| C10—C11 | 1.412 (18) | C64—C65 | 1.33 (2) |
| C10—C31 | 1.51 (2) | C65—C66 | 1.43 (2) |
| C11—N3 | 1.360 (16) | C71—C76 | 1.35 (2) |
| C11—C12 | 1.407 (19) | C71—C72 | 1.392 (18) |
| C12—C13 | 1.336 (17) | C71—P2 | 1.809 (15) |
| C13—C14 | 1.439 (17) | C72—C73 | 1.40 (2) |
| C14—N3 | 1.366 (16) | C73—C74 | 1.35 (2) |
| C14—C15 | 1.378 (17) | C74—C75 | 1.32 (2) |
| C15—C16 | 1.446 (18) | C75—C76 | 1.39 (2) |
| C15—Pd1 | 1.940 (13) | C81—C82 | 1.35 (2) |
| C16—N4 | 1.379 (17) | C81—C93 | 1.39 (2) |
| C16—C17 | 1.422 (19) | C82—C83 | 1.385 (18) |
| C17—C18 | 1.36 (2) | C83—C84 | 1.389 (18) |
| C18—C19 | 1.35 (2) | C84—C92 | 1.404 (17) |
| C19—N4 | 1.381 (17) | C84—P1 | 1.812 (12) |
| C19—C20 | 1.45 (2) | C85—C91 | 1.373 (18) |
| C20—C21 | 1.52 (2) | C85—C86 | 1.400 (17) |
| C21—C26 | 1.26 (2) | C85—P2 | 1.778 (13) |
| C21—C22 | 1.48 (3) | C86—C87 | 1.377 (18) |
| C22—C23 | 1.49 (3) | C87—C88 | 1.370 (19) |
| C23—C24 | 1.46 (4) | C88—C90 | 1.388 (18) |
| C24—C25 | 1.20 (3) | C89—C90 | 1.503 (18) |
| C25—C26 | 1.33 (3) | C89—C93 | 1.51 (2) |
| C31—C36 | 1.37 (2) | C89—C95 | 1.52 (2) |
| C31—C32 | 1.37 (2) | C89—C96 | 1.53 (2) |
| C32—C33 | 1.39 (2) | C90—C91 | 1.386 (17) |
| C33—C34 | 1.32 (2) | C91—O81 | 1.405 (14) |
| C34—C35 | 1.38 (2) | C92—O81 | 1.348 (15) |
| C35—C36 | 1.37 (2) | C92—C93 | 1.414 (17) |
| C41—C46 | 1.381 (18) | O80—P2 | 1.503 (9) |
| C41—C42 | 1.411 (18) | O80—Pd1 | 2.153 (9) |
| C41—P1 | 1.824 (13) | P1—Pd1 | 2.265 (3) |
| C42—C43 | 1.361 (19) | Pd1—Cl1 | 2.366 (3) |
| Angles (º) | | | |
| N1—C1—C20 | 127.8 (17) | C55—C54—C53 | 118.8 (15) |
| N1—C1—C2 | 106 (2) | C54—C55—C56 | 122.0 (14) |
| C20—C1—C2 | 125 (2) | C51—C56—C55 | 120.1 (13) |
| C3—C2—C1 | 106.7 (19) | C66—C61—C62 | 120.1 (14) |
| C2—C3—C4 | 109 (2) | C66—C61—P2 | 121.5 (12) |
| C5—C4—N1 | 127.4 (17) | C62—C61—P2 | 118.2 (11) |
| C5—C4—C3 | 127 (2) | C61—C62—C63 | 120.4 (15) |
| N1—C4—C3 | 105.0 (19) | C64—C63—C62 | 117.5 (17) |
| C4—C5—C6 | 131 (2) | C65—C64—C63 | 122.8 (18) |
| C4—C5—Br5 | 114.1 (15) | C64—C65—C66 | 119.8 (18) |
| C6—C5—Br5 | 115.0 (16) | C61—C66—C65 | 119.2 (16) |
| C5—C6—C7 | 129.3 (19) | C76—C71—C72 | 119.6 (14) |
| C5—C6—N2 | 121.1 (19) | C76—C71—P2 | 123.8 (11) |
| C7—C6—N2 | 109.5 (17) | C72—C71—P2 | 116.6 (11) |
| C8—C7—C6 | 108.5 (19) | C71—C72—C73 | 118.8 (15) |
| C7—C8—C9 | 107.9 (19) | C74—C73—C72 | 119.9 (16) |
| C10—C9—N2 | 125.7 (15) | C75—C74—C73 | 120.4 (18) |
| C10—C9—C8 | 126.1 (17) | C74—C75—C76 | 121.9 (18) |
| N2—C9—C8 | 108.3 (15) | C71—C76—C75 | 119.2 (15) |
| C9—C10—C11 | 126.6 (14) | C82—C81—C93 | 122.0 (14) |
| C9—C10—C31 | 118.6 (13) | C81—C82—C83 | 121.4 (15) |
| C11—C10—C31 | 114.7 (13) | C82—C83—C84 | 119.4 (14) |
| N3—C11—C12 | 108.0 (12) | C83—C84—C92 | 118.6 (11) |
| N3—C11—C10 | 124.5 (13) | C83—C84—P1 | 124.3 (10) |
| C12—C11—C10 | 127.5 (13) | C92—C84—P1 | 117.1 (10) |
| C13—C12—C11 | 107.9 (13) | C91—C85—C86 | 117.5 (12) |
| C12—C13—C14 | 108.6 (13) | C91—C85—P2 | 120.2 (10) |
| N3—C14—C15 | 129.7 (12) | C86—C85—P2 | 122.0 (11) |
| N3—C14—C13 | 105.8 (11) | C87—C86—C85 | 118.3 (14) |
| C15—C14—C13 | 124.4 (12) | C88—C87—C86 | 121.4 (14) |
| C14—C15—C16 | 122.8 (13) | C87—C88—C90 | 122.7 (14) |
| C14—C15—Pd1 | 119.4 (10) | C90—C89—C93 | 107.6 (10) |
| C16—C15—Pd1 | 117.9 (10) | C90—C89—C95 | 106.3 (13) |
| N4—C16—C17 | 109.9 (13) | C93—C89—C95 | 110.2 (12) |
| N4—C16—C15 | 126.5 (12) | C90—C89—C96 | 111.6 (13) |
| C17—C16—C15 | 123.6 (14) | C93—C89—C96 | 112.0 (15) |
| C18—C17—C16 | 103.7 (16) | C95—C89—C96 | 109.0 (13) |
| C19—C18—C17 | 111.8 (15) | C91—C90—C88 | 113.5 (13) |
| C18—C19—N4 | 108.3 (15) | C91—C90—C89 | 119.1 (12) |
| C18—C19—C20 | 128.5 (16) | C88—C90—C89 | 126.8 (12) |
| N4—C19—C20 | 123.2 (17) | C85—C91—C90 | 125.9 (12) |
| C1—C20—C19 | 124.3 (17) | C85—C91—O81 | 114.8 (11) |
| C1—C20—C21 | 121.4 (17) | C90—C91—O81 | 119.2 (12) |
| C19—C20—C21 | 113.8 (18) | O81—C92—C84 | 119.0 (11) |
| C26—C21—C22 | 116 (2) | O81—C92—C93 | 119.8 (12) |
| C26—C21—C20 | 132.4 (19) | C84—C92—C93 | 121.1 (13) |
| C22—C21—C20 | 110.5 (18) | C81—C93—C92 | 116.8 (14) |
| C21—C22—C23 | 113 (2) | C81—C93—C89 | 124.3 (12) |
| C24—C23—C22 | 117 (2) | C92—C93—C89 | 118.7 (13) |
| C25—C24—C23 | 119 (3) | C1—N1—C4 | 112.2 (16) |
| C24—C25—C26 | 121 (3) | C9—N2—C6 | 105.6 (15) |
| C21—C26—C25 | 129 (2) | C11—N3—C14 | 109.6 (11) |
| C36—C31—C32 | 116.6 (16) | C16—N4—C19 | 106.1 (13) |
| C36—C31—C10 | 120.0 (16) | P2—O80—Pd1 | 125.7 (5) |
| C32—C31—C10 | 123.4 (15) | C92—O81—C91 | 116.8 (10) |
| C31—C32—C33 | 122.8 (19) | C51—P1—C84 | 105.9 (6) |
| C34—C33—C32 | 119 (2) | C51—P1—C41 | 101.8 (6) |
| C33—C34—C35 | 120 (2) | C84—P1—C41 | 104.0 (6) |
| C36—C35—C34 | 120.3 (19) | C51—P1—Pd1 | 114.6 (4) |
| C31—C36—C35 | 121.2 (19) | C84—P1—Pd1 | 111.9 (4) |
| C46—C41—C42 | 118.7 (13) | C41—P1—Pd1 | 117.2 (4) |
| C46—C41—P1 | 119.9 (11) | O80—P2—C85 | 115.0 (6) |
| C42—C41—P1 | 121.0 (10) | O80—P2—C71 | 111.7 (6) |
| C43—C42—C41 | 120.1 (14) | C85—P2—C71 | 110.4 (6) |
| C42—C43—C44 | 120.8 (16) | O80—P2—C61 | 112.5 (6) |
| C45—C44—C43 | 119.9 (16) | C85—P2—C61 | 101.6 (6) |
| C44—C45—C46 | 121.1 (16) | C71—P2—C61 | 104.8 (6) |
| C41—C46—C45 | 119.4 (16) | C15—Pd1—O80 | 175.6 (4) |
| C56—C51—C52 | 117.6 (12) | C15—Pd1—P1 | 91.1 (4) |
| C56—C51—P1 | 122.1 (10) | **O80—Pd1—P1** | **93.2 (2)** |
| C52—C51—P1 | 120.3 (9) | C15—Pd1—Cl1 | 86.4 (4) |
| C53—C52—C51 | 120.7 (12) | O80—Pd1—Cl1 | 89.3 (2) |
| C52—C53—C54 | 120.7 (14) | P1—Pd1—Cl1 | 176.78 (14) |
| Torsion angles (º) | | | |
| N1—C1—C2—C3 | -5 (2) | C73—C74—C75—C76 | 2 (3) |
| C20—C1—C2—C3 | -177.1 (18) | C72—C71—C76—C75 | -1 (2) |
| C1—C2—C3—C4 | 7 (2) | P2—C71—C76—C75 | -179.5 (14) |
| C2—C3—C4—C5 | 171 (2) | C74—C75—C76—C71 | -2 (3) |
| C2—C3—C4—N1 | -6 (2) | C93—C81—C82—C83 | -3 (2) |
| N1—C4—C5—C6 | -9 (4) | C81—C82—C83—C84 | 2 (2) |
| C3—C4—C5—C6 | 175 (2) | C82—C83—C84—C92 | 4 (2) |
| N1—C4—C5—Br5 | 173.4 (15) | C82—C83—C84—P1 | -172.7 (11) |
| C3—C4—C5—Br5 | -3 (3) | C91—C85—C86—C87 | 2 (2) |
| C4—C5—C6—C7 | 177 (2) | P2—C85—C86—C87 | -170.7 (11) |
| Br5—C5—C6—C7 | -5 (3) | C85—C86—C87—C88 | 2 (2) |
| C4—C5—C6—N2 | -5 (3) | C86—C87—C88—C90 | -1 (2) |
| Br5—C5—C6—N2 | 173.1 (12) | C87—C88—C90—C91 | -5 (2) |
| C5—C6—C7—C8 | 174.8 (19) | C87—C88—C90—C89 | 166.4 (14) |
| N2—C6—C7—C8 | -4 (2) | C93—C89—C90—C91 | -39.2 (18) |
| C6—C7—C8—C9 | 1 (2) | C95—C89—C90—C91 | 78.8 (16) |
| C7—C8—C9—C10 | -179.2 (17) | C96—C89—C90—C91 | -162.5 (15) |
| C7—C8—C9—N2 | 2 (2) | C93—C89—C90—C88 | 149.9 (15) |
| N2—C9—C10—C11 | 7 (3) | C95—C89—C90—C88 | -92.1 (18) |
| C8—C9—C10—C11 | -172.1 (16) | C96—C89—C90—C88 | 27 (2) |
| N2—C9—C10—C31 | -169.8 (15) | C86—C85—C91—C90 | -9 (2) |
| C8—C9—C10—C31 | 12 (3) | P2—C85—C91—C90 | 164.4 (11) |
| C9—C10—C11—N3 | -1 (2) | C86—C85—C91—O81 | 175.8 (11) |
| C31—C10—C11—N3 | 175.1 (13) | P2—C85—C91—O81 | -11.0 (16) |
| C9—C10—C11—C12 | 176.9 (15) | C88—C90—C91—C85 | 10 (2) |
| C31—C10—C11—C12 | -7 (2) | C89—C90—C91—C85 | -162.2 (14) |
| N3—C11—C12—C13 | 0.5 (17) | C88—C90—C91—O81 | -174.9 (12) |
| C10—C11—C12—C13 | -178.0 (14) | C89—C90—C91—O81 | 13.0 (19) |
| C11—C12—C13—C14 | -0.2 (17) | C83—C84—C92—O81 | 170.5 (12) |
| C12—C13—C14—N3 | -0.1 (16) | P1—C84—C92—O81 | -12.7 (15) |
| C12—C13—C14—C15 | 179.0 (13) | C83—C84—C92—C93 | -9.8 (18) |
| N3—C14—C15—C16 | -1 (2) | P1—C84—C92—C93 | 167.1 (10) |
| C13—C14—C15—C16 | -179.8 (13) | C82—C81—C93—C92 | -3 (2) |
| N3—C14—C15—Pd1 | 178.3 (11) | C82—C81—C93—C89 | -178.0 (14) |
| C13—C14—C15—Pd1 | -0.6 (19) | O81—C92—C93—C81 | -171.2 (12) |
| C14—C15—C16—N4 | -1 (2) | C84—C92—C93—C81 | 9.0 (19) |
| Pd1—C15—C16—N4 | 179.3 (10) | O81—C92—C93—C89 | 4.5 (19) |
| C14—C15—C16—C17 | 177.2 (14) | C84—C92—C93—C89 | -175.2 (12) |
| Pd1—C15—C16—C17 | -2.1 (18) | C90—C89—C93—C81 | -153.9 (14) |
| N4—C16—C17—C18 | 2.6 (17) | C95—C89—C93—C81 | 90.6 (18) |
| C15—C16—C17—C18 | -176.3 (13) | C96—C89—C93—C81 | -31 (2) |
| C16—C17—C18—C19 | -5.0 (19) | C90—C89—C93—C92 | 30.7 (18) |
| C17—C18—C19—N4 | 6 (2) | C95—C89—C93—C92 | -84.8 (15) |
| C17—C18—C19—C20 | -176.6 (15) | C96—C89—C93—C92 | 153.6 (13) |
| N1—C1—C20—C19 | 17 (3) | C20—C1—N1—C4 | 173.2 (18) |
| C2—C1—C20—C19 | -172.5 (16) | C2—C1—N1—C4 | 1 (2) |
| N1—C1—C20—C21 | -171.4 (17) | C5—C4—N1—C1 | -174 (2) |
| C2—C1—C20—C21 | -1 (3) | C3—C4—N1—C1 | 3 (2) |
| C18—C19—C20—C1 | 176.8 (17) | C10—C9—N2—C6 | 177.1 (16) |
| N4—C19—C20—C1 | -6 (3) | C8—C9—N2—C6 | -4.1 (18) |
| C18—C19—C20—C21 | 5 (2) | C5—C6—N2—C9 | -173.8 (16) |
| N4—C19—C20—C21 | -178.0 (14) | C7—C6—N2—C9 | 5 (2) |
| C1—C20—C21—C26 | -111 (3) | C12—C11—N3—C14 | -0.6 (15) |
| C19—C20—C21—C26 | 61 (3) | C10—C11—N3—C14 | 178.0 (13) |
| C1—C20—C21—C22 | 55 (2) | C15—C14—N3—C11 | -178.6 (14) |
| C19—C20—C21—C22 | -132.4 (19) | C13—C14—N3—C11 | 0.4 (15) |
| C26—C21—C22—C23 | -15 (4) | C17—C16—N4—C19 | 0.7 (15) |
| C20—C21—C22—C23 | 177 (2) | C15—C16—N4—C19 | 179.5 (13) |
| C21—C22—C23—C24 | 1 (4) | C18—C19—N4—C16 | -3.7 (17) |
| C22—C23—C24—C25 | 17 (6) | C20—C19—N4—C16 | 178.4 (14) |
| C23—C24—C25—C26 | -21 (5) | C84—C92—O81—C91 | 144.7 (11) |
| C22—C21—C26—C25 | 14 (4) | C93—C92—O81—C91 | -35.1 (16) |
| C20—C21—C26—C25 | 180 (2) | C85—C91—O81—C92 | -158.0 (11) |
| C24—C25—C26—C21 | 5 (5) | C90—C91—O81—C92 | 26.3 (16) |
| C9—C10—C31—C36 | 74 (2) | C56—C51—P1—C84 | 19.0 (13) |
| C11—C10—C31—C36 | -102.3 (17) | C52—C51—P1—C84 | -158.6 (10) |
| C9—C10—C31—C32 | -108 (2) | C56—C51—P1—C41 | -89.5 (12) |
| C11—C10—C31—C32 | 76 (2) | C52—C51—P1—C41 | 92.9 (11) |
| C36—C31—C32—C33 | 1 (3) | C56—C51—P1—Pd1 | 143.0 (10) |
| C10—C31—C32—C33 | -177.2 (17) | C52—C51—P1—Pd1 | -34.6 (11) |
| C31—C32—C33—C34 | 1 (3) | C83—C84—P1—C51 | -97.9 (12) |
| C32—C33—C34—C35 | -4 (3) | C92—C84—P1—C51 | 85.5 (10) |
| C33—C34—C35—C36 | 4 (3) | C83—C84—P1—C41 | 9.0 (13) |
| C32—C31—C36—C35 | -1 (3) | C92—C84—P1—C41 | -167.6 (10) |
| C10—C31—C36—C35 | 177.4 (16) | C83—C84—P1—Pd1 | 136.5 (10) |
| C34—C35—C36—C31 | -2 (3) | C92—C84—P1—Pd1 | -40.1 (11) |
| C46—C41—C42—C43 | 0 (2) | C46—C41—P1—C51 | -18.9 (12) |
| P1—C41—C42—C43 | 173.1 (10) | C42—C41—P1—C51 | 168.2 (10) |
| C41—C42—C43—C44 | 0 (2) | C46—C41—P1—C84 | -128.9 (11) |
| C42—C43—C44—C45 | 0 (3) | C42—C41—P1—C84 | 58.2 (12) |
| C43—C44—C45—C46 | 0 (3) | C46—C41—P1—Pd1 | 106.9 (11) |
| C42—C41—C46—C45 | 0 (2) | C42—C41—P1—Pd1 | -65.9 (11) |
| P1—C41—C46—C45 | -173.3 (13) | Pd1—O80—P2—C85 | 17.3 (8) |
| C44—C45—C46—C41 | 0 (3) | Pd1—O80—P2—C71 | -109.6 (7) |
| C56—C51—C52—C53 | 4.0 (19) | Pd1—O80—P2—C61 | 132.9 (6) |
| P1—C51—C52—C53 | -178.2 (10) | C91—C85—P2—O80 | 54.9 (12) |
| C51—C52—C53—C54 | -2 (2) | C86—C85—P2—O80 | -132.3 (11) |
| C52—C53—C54—C55 | 2 (2) | C91—C85—P2—C71 | -177.6 (11) |
| C53—C54—C55—C56 | -3 (2) | C86—C85—P2—C71 | -4.8 (14) |
| C52—C51—C56—C55 | -5 (2) | C91—C85—P2—C61 | -66.8 (12) |
| P1—C51—C56—C55 | 177.1 (11) | C86—C85—P2—C61 | 106.0 (12) |
| C54—C55—C56—C51 | 5 (2) | C76—C71—P2—O80 | -173.6 (13) |
| C66—C61—C62—C63 | -5 (2) | C72—C71—P2—O80 | 7.6 (13) |
| P2—C61—C62—C63 | 178.7 (12) | C76—C71—P2—C85 | 57.1 (15) |
| C61—C62—C63—C64 | 3 (2) | C72—C71—P2—C85 | -121.8 (11) |
| C62—C63—C64—C65 | 1 (3) | C76—C71—P2—C61 | -51.6 (15) |
| C63—C64—C65—C66 | -2 (3) | C72—C71—P2—C61 | 129.6 (11) |
| C62—C61—C66—C65 | 4 (3) | C66—C61—P2—O80 | -156.9 (13) |
| P2—C61—C66—C65 | 179.7 (14) | C62—C61—P2—O80 | 18.9 (14) |
| C64—C65—C66—C61 | 0 (3) | C66—C61—P2—C85 | -33.4 (15) |
| C76—C71—C72—C73 | 3 (2) | C62—C61—P2—C85 | 142.4 (12) |
| P2—C71—C72—C73 | -178.1 (11) | C66—C61—P2—C71 | 81.6 (15) |
| C71—C72—C73—C74 | -3 (2) | C62—C61—P2—C71 | -102.6 (12) |
| C72—C73—C74—C75 | 0 (3) |  |  |


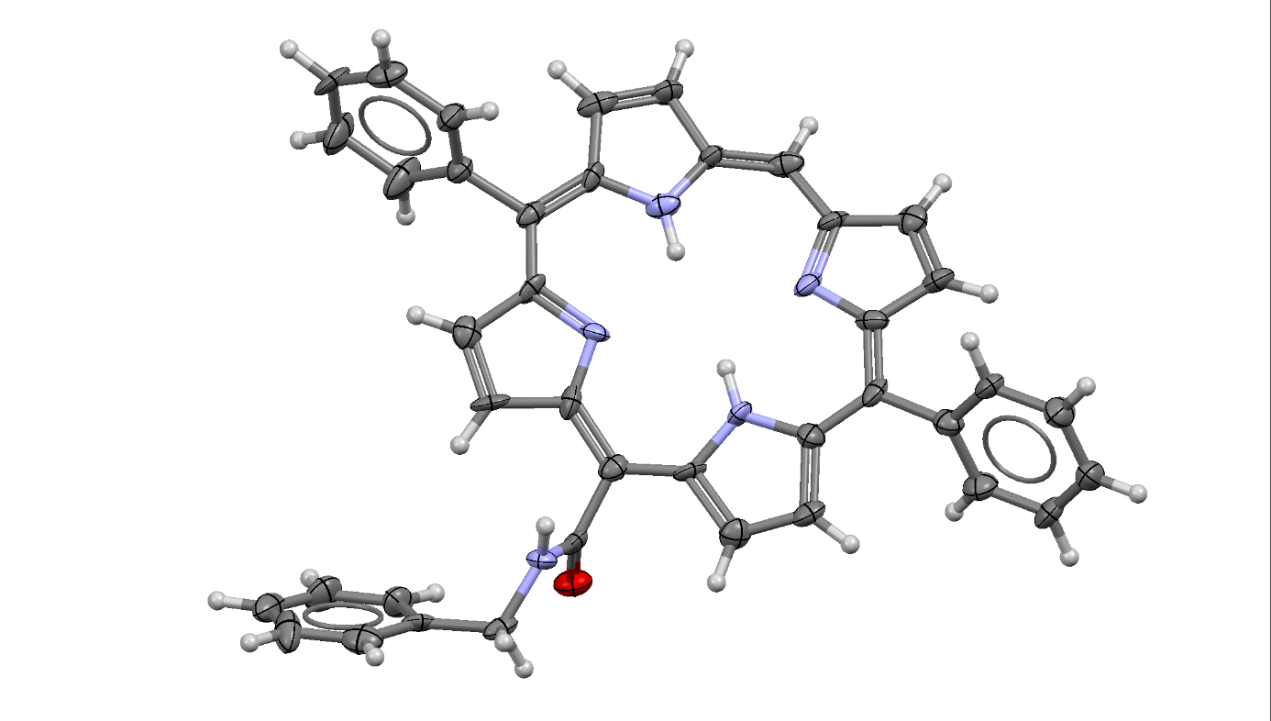


*Figure S1.* ORTEP view of **DPP-Bn** at 40% probability level


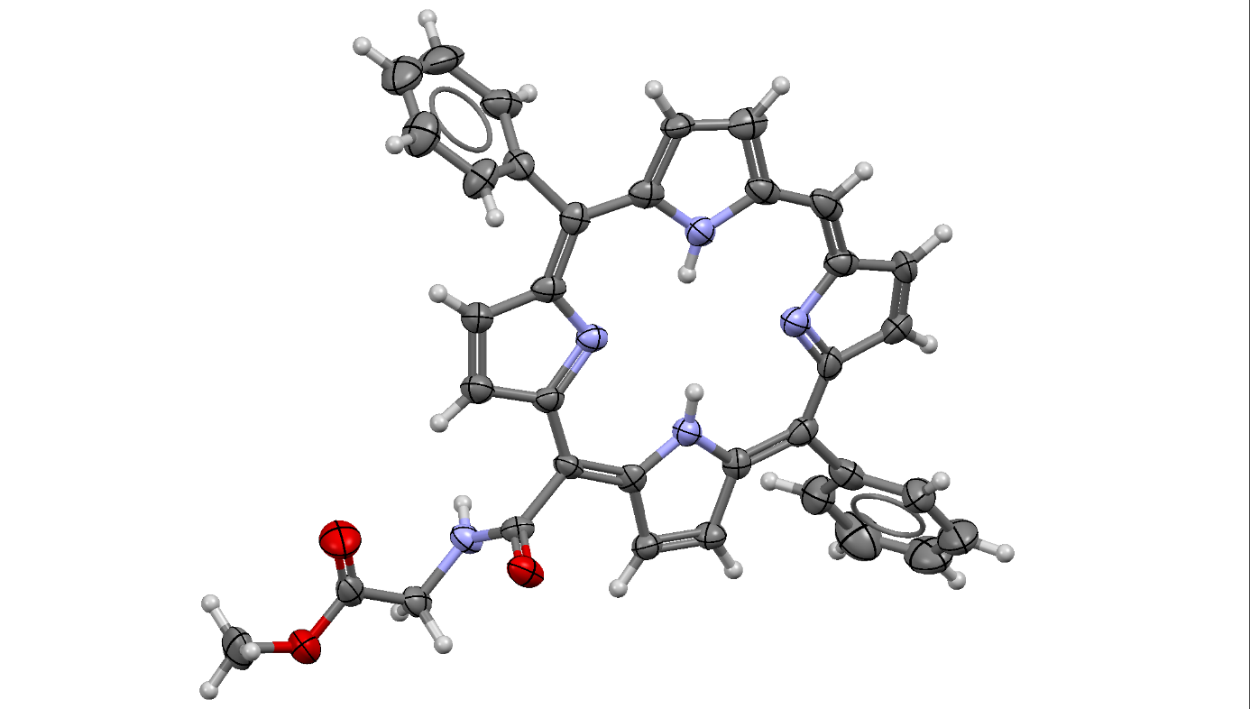


*Figure S2.* ORTEP view of **DPP-Gly** at 40% probability level. Chloroform solvent molecule is omitted for clarity.


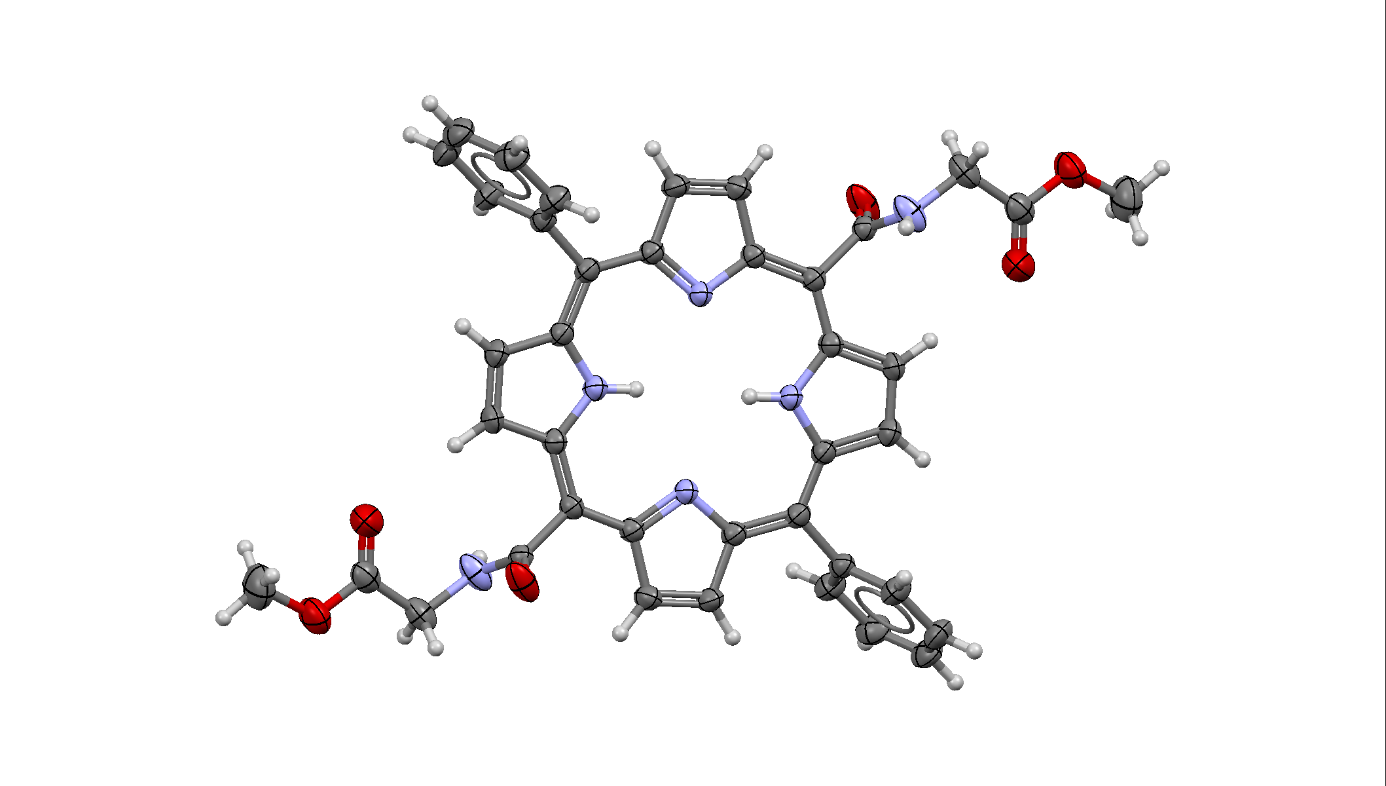


*Figure S3.* ORTEP view of **DPP-Gly2** at 40% probability level, solvent chloroform molecule is omitted for clarity


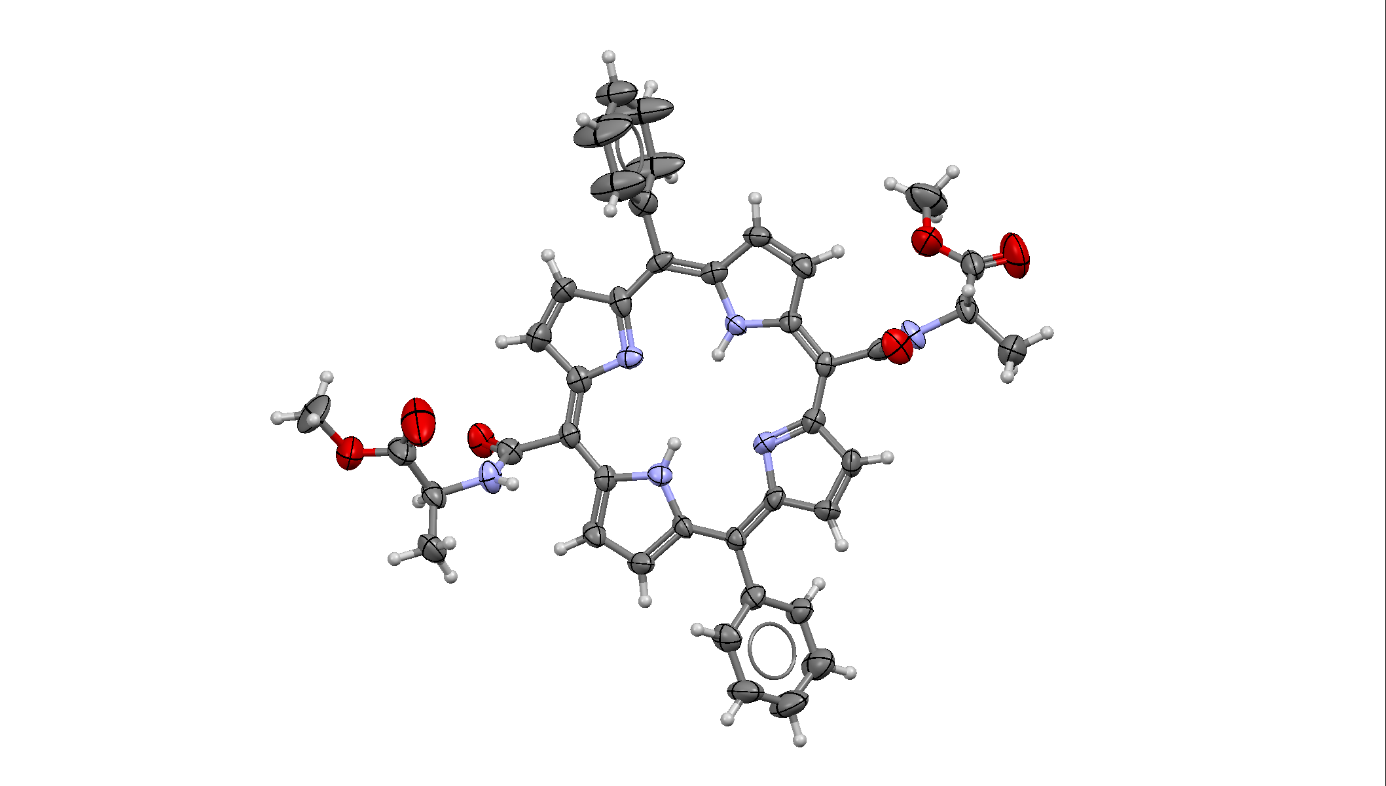


*Figure S4.* ORTEP view of **DPP-Ala2** at 40% probability level.

The compound of **DPP-Ala2** crystallised in orthorhombic chiral space group of No.19 (P212121) as it was expected as we have pure enantiomer. The synthesis started from the natural amino acid and the configuration of the stereogenic centers were preserved.

*Palladium-catalysed aminocarbonylation of* **DPPBr2** (**1**)

In a 100 mL three-necked round bottom flask, equipped with a magnetic stirrer bar, a gas inlet and a reflux condenser with a balloon (filled with argon) at the top, Pd(OAc)2 (2.8 mg, 0.012 mmol), xantphos (7.1 mg, 0.012 mmol), 5,15-dibromo-10,20-diphenylporphyrin (**1**) (62 mg, 0.1 mmol) and glycine methyl ester hydrochloride (27.6 mg, 0.22 mmol) were dissolved in dry toluene (8 mL) under argon atmosphere. Triethylamine (0.1 mL, 0.72 mmol) was added to the reaction mixture and the argon atmosphere was changed to carbon monoxide by using a vacuum-carbon monoxide line. The reaction was conducted for 24 h upon stirring at 70 ˚C and analyzed by thin-layer chromatography. The reaction mixture was then evaporated to dryness. The crude residue was subjected to column chromatography (Silica gel 60 (Merck), 0.063-0.200 mm. The isolated product was recrystallized from mixture of chloroform and n-hexane.

*Synthesis of* ***2***

Pd(OAc)2 (11.2 mg, 0.05 mmol), **DPPBr2** **1** (31.0 mg, 0.05 mmol) and Xantphos (28.9 mg, 0.05 mmol) were dissolved in dry toluene (10 mL) under argon atmosphere in a 100 mL three-necked round bottom flask equipped with a magnetic stirrer bar, a gas inlet and a reflux condenser with a balloon (filled with argon) at the top. The reaction was conducted for 24 hours upon stirring at 70 ˚C. After cooling to room temperature, the solution was filtered to remove some Pd metal. The filtrate was then concentrated and evaporated to dryness and recrystallised for the mixture of chloroform/hexane (70:30).

***Characterization of 2***


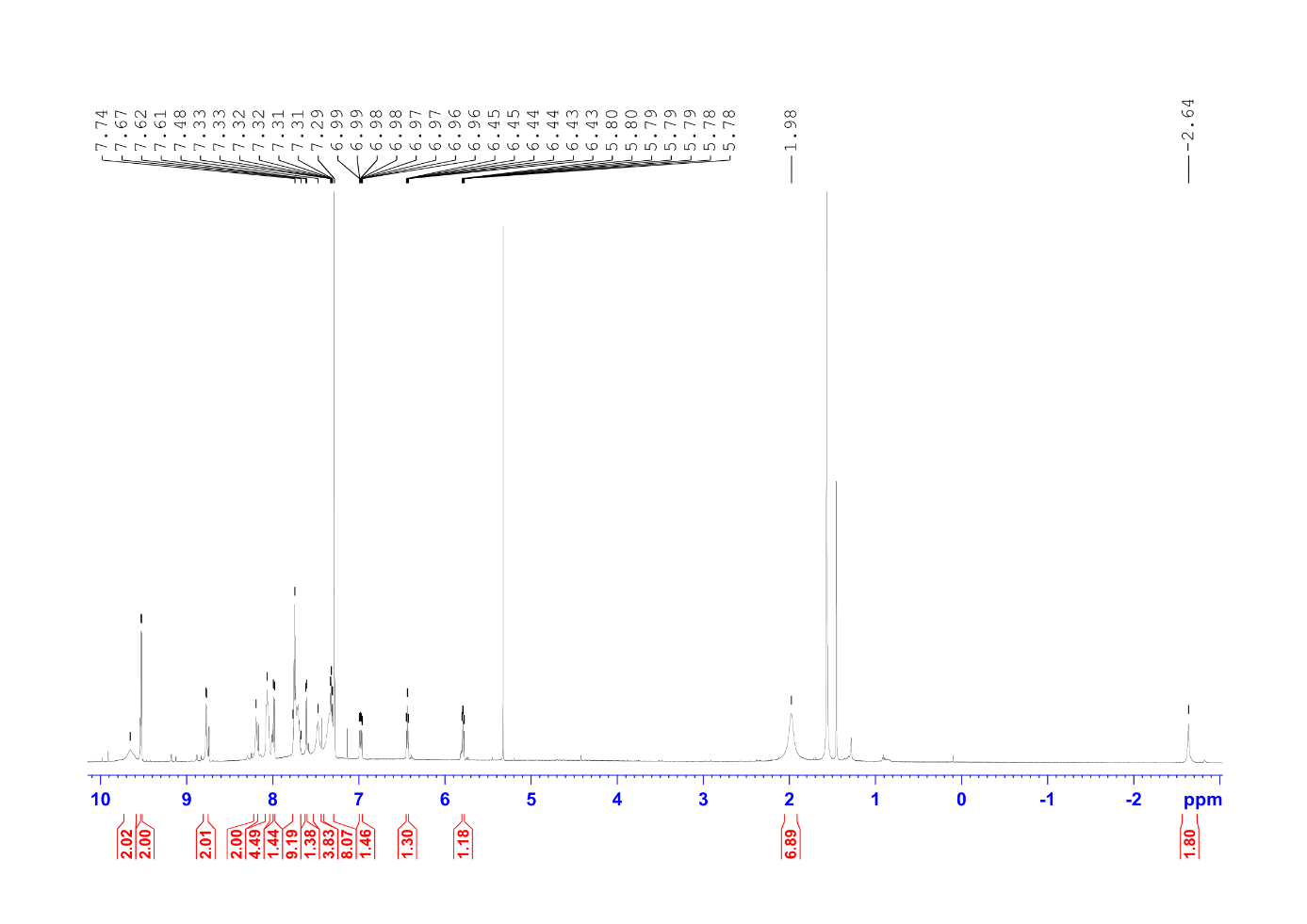
δH (600 MHz, CDCl3): 9.66 (brs, 2H), 9.53 (2H, d, *J*= 4.8 Hz), 8.77 (2H, d, *J*= 4.8 Hz), 8.20 (2H, brd, *J*= 4.0 Hz), 8.06 (4H, brs), 7.99 (1H, dd, *J*= 8.0 Hz, 1.0 Hz), 7.76-7.65 (8H, m), 7.61 (1H, d, *J*= 7.6 Hz), 7.52-7.31 (12H, m, Ar), 7.15-7.00 (6H, m, Ar), 7.32 (1H, ddd, *J*= 7.7 Hz, 7.5 Hz, 2.7 Hz), 6.98 (1H, ddd, *J*= 13.8 Hz, 7.2 Hz, 1.2 Hz), 6.43 (1H, dt, *J*= 11.2 Hz, 1.0Hz), 5.79 (1H, ddd, *J*= 9.5 Hz, 7.6 Hz, 1.4 Hz), 1.97 (6H, brs, -(CH3)2 ), -2.63 (2H, brs, NH).

*Figure S5.* 1H-NMR spectrum of **2**

δP (236 MHz, CDCl3): 42.09 (P=O), 9.82 (P).


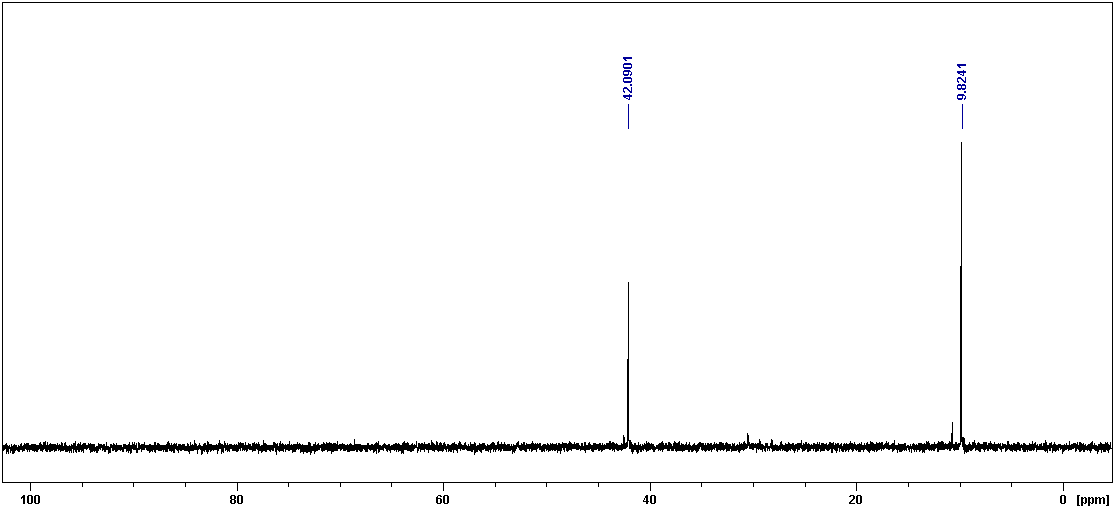


*Figure S6.* 31P-NMR spectrum of **2**


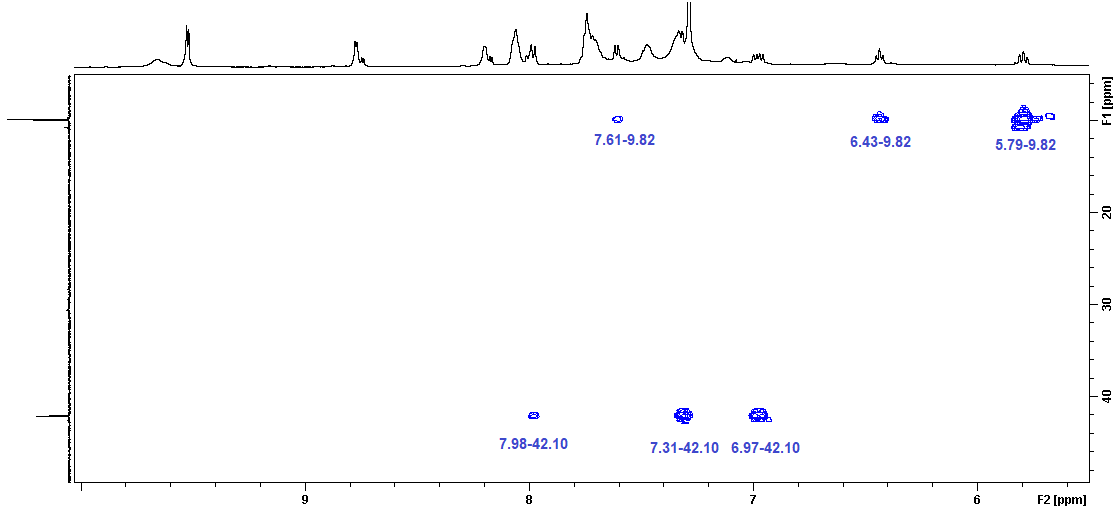


*Figure S7.* 1H-31P HMBC correlation spectrum of **2**

**
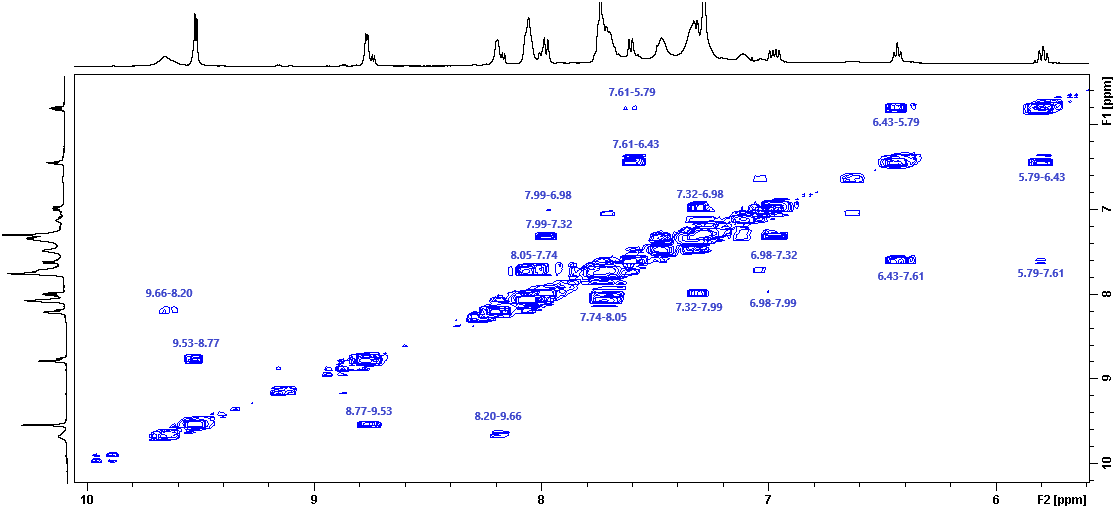
**

*Figure S8.* 1H-1H COSY correlation spectrum of **2** (represent only the aromatic region)

**
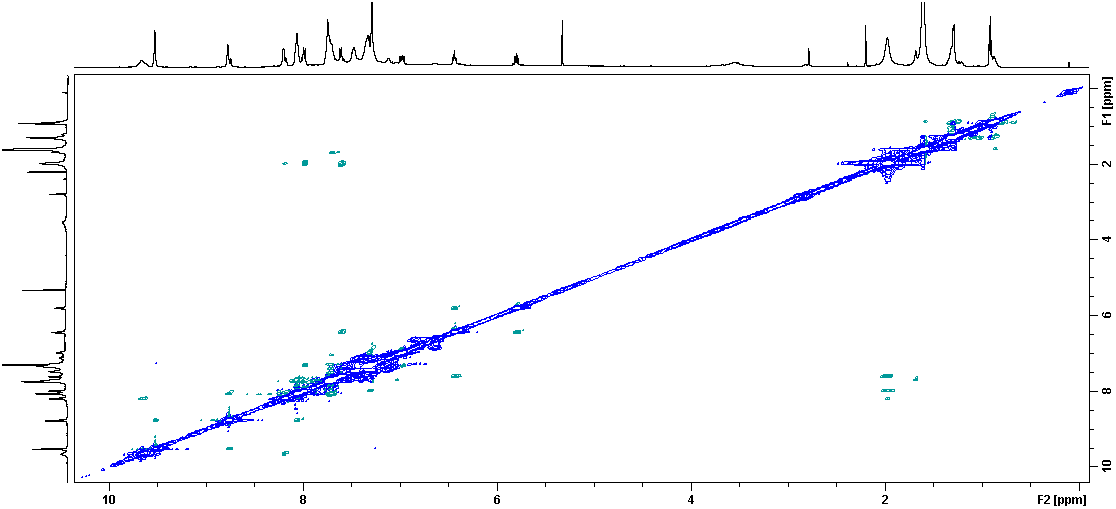
**

*Figure S9.* 1H-1H ROESY correlation spectrum of **2**

**
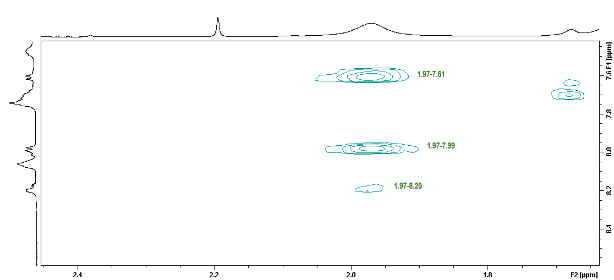

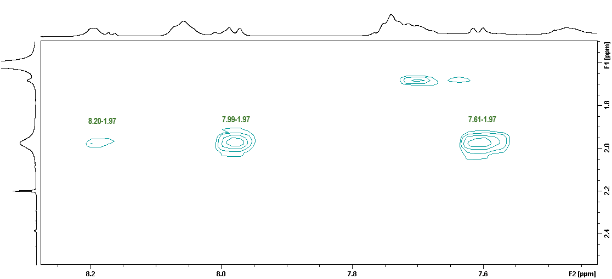
**

*Figure S10.* 1H-1H ROESY correlation spectrum of **2 (**represent only the regions of the CH3-group)

**
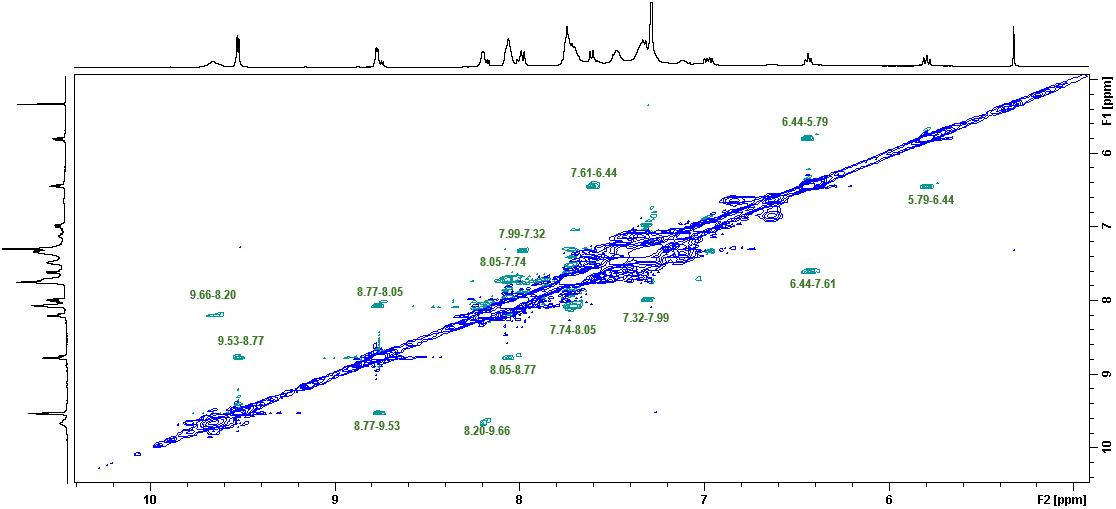
**

*Figure S11.* 1H-1H ROESY correlation spectrum of **2** (represent only the aromatic region)

*Figure S12.* Representation of the structure of **2** based ont he NMR spectra (from *figure S5.* to *figure S11.*)

HRMS of **2** (ESI-Q-TOF, positive ions): m/z = 1321.1045 (calculated for [C71H53N4O2Br2P2Pd]+ [M+H]+ 1321.1046).

HRMS isotope pattern (ESI-Q-TOF, positive ions) m/z (rel. int.): 1317.1086 (7.7283), 1318.1074 (18.4319), 1319.1057 (45.3070), 1320.1065 (59.7200), 1321.1045 (100), 1322.1064 (75.9034), 1323.1045 (96.3139), 1324.1071 (58.7971), 1325.1057 (53.7811), 1326.1072 (29.7146), 1327.1063 (16.7584), 1328.1064 (7.5528), 1329.1095 (2.9668). Calculated for [C71H53N4O2Br2P2Pd]+ [M+H]+: 1317.1059 (9.5268), 1318.1043 (23.1733), 1319.1026 (50.2580), 1320.1063 (64.1561), 1321.1046 (100), 1322.1082 (77.2017), 1323.1066 (96.4461), 1324.1050 (59.9008), 1325.1033 (55.0811), 1326.1069 (32.0974), 1327.1053 (19.2083), 1328.1089 (9.0259), 1329.1073 (2.9855).

HRMS (ESI-Q-TOF, positive ions): m/z = 1241.1793 (calculated for [C71H53N4O2BrP2Pd]+ [(M-Br)+H]+ 1241.1771).

HRMS isotope pattern (ESI-Q-TOF, positive ions) m/z (rel. int.): 1235.1802 (0.7896), 1236.1844 (0.6151), 1237.1781 (10.1463), 1238.1808 (31.5933), 1239.1799 (65.5112), 1240.1808 (67.5792), 1241.1793 (100), 1242.1822 (61.6214), 1243.1813 (66.7273), 1244.1832 (39.7382), 1245.1831 (26.8515), 1246.1834 (12.9573), 1247.1855 (4.5986), 1248.1892 (1.4804). Calculated for [C71H53N4O2BrP2Pd]+ [(M-Br)+H]+: 1235.1812 (1.2290), 1236.1830 (0.9709), 1237.1799 (14.9711), 1238.1817 (38.4464), 1239.1785 (71.1990), 1240.1803 (71.4876), 1241.1771 (100), 1242.1839 (61.9772), 1243.1808 (66.9557), 1244.1826 (41.0248), 1245.1794 (28.4146), 1246.1812 (14.5503), 1247.1830 (4.9784), 1248.1897 (1.2304).

*Figure S13.* Representation of the isotope pattern of the [M+H]+ molecule cation, formed for **2**

*Figure S14.* Representation of the isotope pattern of the [M-Br+H]+ molecule cation, formed for **2**

*Figure S15.* Fragmentation of the [M+H]+ molecule cation, formed from **2**

IR (ATR, ν (cm-1)): 3055, 2965, 1715, 1595, 1572, 1436, 1404, 1326, 1228, 1145, 963, 844, 789, 747, 720, 693, 530, 501, 465.

***Characterization of 3***

δP (236 MHz, CDCl3): 42.08 (P=O), 9.26 (P).

HRMS (ESI-Q-TOF, positive ions): m/z = 1277.1554 (calculated for [C71H53N4O2BrClP2Pd]+ [M+H]+ 1277.1547).

HRMS isotope pattern (ESI-Q-TOF, positive ions) m/z (rel. int.): 1273.1531 (9.5293), 1274.1564 (26.1194), 1275.1563 (56.7457), 1276.1568 (64.6287), 1277.1554 (100), 1278.1572 (67.6206), 1279.1559 (79.4592), 1280.1579 (47.2268), 1281.1568 (37.8421), 1282.1562 (19.9936), 1283.1554 (10.0540), 1284.1535 (4.3363). Calculated for [C71H53N4O2BrClP2Pd]+ [(M+H]+: 1273.1530 (12.4200), 1274.1582 (31.5109), 1275.1536 (61.4540), 1276.1542 (67.6989), 1277.1547 (100), 1278.1552 (68.9696), 1279.1557 (80.3957), 1280.1563 (49.5136), 1281.1568 (40.3785), 1282.1573 (22.4464), 1283.1578 (11.3438), 1284.1583 (4.7391).

HRMS (ESI-Q-TOF, positive ions): m/z = 1241.1793 (calculated for [C71H53N4O2BrP2Pd]+ [(M-Cl)+H]+ 1241.1771).

HRMS isotope pattern (ESI-Q-TOF, positive ions) m/z (rel. int.): 1235.1802 (0.7896), 1236.1844 (0.6151), 1237.1781 (10.1463), 1238.1808 (31.5933), 1239.1799 (65.5112), 1240.1808 (67.5792), 1241.1793 (100), 1242.1822 (61.6214), 1243.1813 (66.7273), 1244.1832 (39.7382), 1245.1831 (26.8515), 1246.1834 (12.9573), 1247.1855 (4.5986), 1248.1892 (1.4804). Calculated for [C71H53N4O2BrP2Pd]+ [(M-Cl)+H]+: 1235.1812 (1.2290), 1236.1830 (0.9709), 1237.1799 (14.9711), 1238.1817 (38.4464), 1239.1785 (71.1990), 1240.1803 (71.4876), 1241.1771 (100), 1242.1839 (61.9772), 1243.1808 (66.9557), 1244.1826 (41.0248), 1245.1794 (28.4146), 1246.1812 (14.5503), 1247.1830 (4.9784), 1248.1897 (1.2304).

*Figure S16.* Fragmentation of the [M+H]+ molecule cation, formed from **3**

*Figure S17.* Representation of the isotope pattern of the [M-Cl+H]+ molecule cation, formed for **3**

*Figure S19.* Fragmentation of the [M+H]+ molecule cation, formed from **3**
